# Consequences of the loss of catalytic triads in chloroplast CLPPR protease core complexes in vivo

**DOI:** 10.1002/pld3.86

**Published:** 2018-10-25

**Authors:** Jui‐Yun Rei Liao, Giulia Friso, Jitae Kim, Klaas J. van Wijk

**Affiliations:** ^1^ Section of Plant Biology School of Integrative Plant Sciences (SIPS) Cornell University Ithaca New York

**Keywords:** catalytic triad, chloroplast, CLP protease, CLPP3, CLPP5, substrates

## Abstract

The essential chloroplast CLP protease system consists of a tetradecameric proteolytic core with catalytic P (P1, 3–6) and non‐catalytic R (R1–4) subunits, CLP chaperones and adaptors. The chloroplast CLP complex has a total of ten catalytic sites,but it is not known how many of these catalytic sites can be inactivated before plants lose viability. Here we show that CLPP3 and the catalytically inactive variant CLPP3S164A fully complement the developmental arrest of the *clpp3‐1* null mutant, even under environmental stress. In contrast, whereas the inactive variant CLPP5S193A assembled into the CLP core, it cannot rescue the embryo lethal phenotype of the *clpp5‐1* null mutant. This shows that CLPP3 makes a unique structural contribution but its catalytic site is dispensable, whereas the catalytic activity of CLPP5 is essential. Mass spectrometry of affinity‐purified CLP cores of the complemented lines showed highly enriched CLP cores. Other chloroplast proteins were co‐purified with the CLP cores and are candidate substrates. A strong overlap of co‐purified proteins between the CLP core complexes with active and inactive subunits indicates that CLP cores with reduced number of catalytic sites do not over‐accumulate substrates, suggesting that the bottle‐neck for degradation is likely substrate recognition and unfolding by CLP adaptors and chaperones, upstream of the CLP core.

## 1 INTRODUCTION

ATP‐dependent Clp proteases are present in bacteria, as well as mitochondria and plastids (organelles of bacterial origin), where they regulate accumulation levels of a broad range of substrates (Alexopoulos, Guarne, & Ortega, 2012; Liu, Ologbenla, & Houry, 2014; Nishimura, Kato, & Sakamoto, 2017; Nishimura & van Wijk, 2015; Sauer & Baker, 2011). The first step in the CLP degradation process requires the recognition of substrates by the CLP AAA+ chaperones, possibly aided by specific adaptors (also named recognins) that recognize and deliver specific substrates (Kuhlmann & Chien, 2017; Mahmoud & Chien, 2018). The ATP‐dependent CLP chaperones then dock onto CLP protease core complexes consisting of two stacked heptameric rings, and unfold and direct substrates into the CLP protease complex (Olivares, Baker, & Sauer, 2018). The substrates are cleaved within the CLP protease complex and short (~7–10 amino acids) peptide fragments are released through lateral pores in the CLP protease (Alexopoulos et al., 2012; Liu et al., 2014; Peltier et al., 2004). To ensure optimal cellular levels of functional proteins and to remove unwanted proteins while avoiding uncontrolled degradation, substrate recognition and delivery by the adaptors and chaperones must be tightly regulated and depends also on the availability and exposure of degrons in the substrates.

The CLP proteolytic core *in Escherichia coli* consists of 14 identical CLPP subunits that belong to the family of serine‐type proteases (Wang, Hartling, & Flanagan, 1997). The photosynthetic bacterium *Synechococcus elongatus* contain three different CLPP proteins, and one CLPR proteins (Schelin, Lindmark, & Clarke, 2002), which is structurally similar to CLPP but lacks the catalytic residues for peptide bond hydrolysis. These cyanobacterial proteins assemble into an essential CLPR/P3 complex (identical heptameric rings with P3:R in a 3:4 ratio) and a non‐essential CLPP1/P2 complex (Andersson et al., 2009; Stanne, Pojidaeva, Andersson, & Clarke, 2007). The apicoplast of *Plasmodium falsiparum* accumulates both a single CLPP and CLPR protein but they do appear to form separate homo‐oligomers (El Bakkouri et al., 2013). Compared to these homologs, the chloroplast CLP system is far more diversified and complex (Nishimura & van Wijk, 2015; Olinares, Kim, Davis, & van Wijk, 2011; Yu & Houry, 2007).

The chloroplast CLP protease complex in Arabidopsis contains five different CLPP subunits namely plastid‐encoded CLPP1 and the nuclear‐encoded CLPP3‐6, and four different non‐catalytic CLPR subunits CLPR1‐4. The five CLPP subunits with conserved catalytic sites accumulate in a 3:1:2:3:1 ratio for P1:P3:P4:P5:P6, whereas the non‐catalytic CLPR subunits are each present in one copy. Three copies of CLPP1 and the four CLPR subunits form the heptameric R‐ring, whereas CLPP3‐6 form the heptameric P‐ring. Therefore the chloroplast CLP complex has a total of ten catalytic sites, of which seven in the P‐ring and only three in the R‐ring. It is not known how many and which of these catalytic sites can be inactivated before plants lose viability. The chloroplast CLP core also associates with two plant specific proteins ClpT1 and ClpT2. These CLPT subunits likely function in CLPPR core formation, stabilization and activation (Kim et al., 2015; Sjogren & Clarke, 2011).

Arabidopsis CLPP and CLPR proteins are not very conserved to each other at the primary sequence level (24–48% identities between CLPPs; 28–38% identities between CLPRs). Severe phenotypes were found in Arabidopsis *clpp* and *clpr* mutants and in tobacco RNAi lines (Moreno et al., 2017) at various developmental stages (Kim et al., 2009, 2013; Koussevitzky et al., 2007; Rudella, Friso, Alonso, Ecker, & van Wijk, 2006; Zheng, MacDonald, Sutinen, Hurry, & Clarke, 2006). For example, null mutants in *CLPP3* (*clpp3‐1*) are seedling lethal and are arrested in the cotyledon stage. However, the addition of sucrose breaks this developmental arrest and plants form pale‐green leaves and eventually flower and produce seed (Kim et al., 2013). In contrast, null mutants in *CLPP5* (*clpp5‐1*) are embryo lethal (Kim et al., 2009). These phenotypes showed that both CLP3 and CLP5 are required for plant development, and we speculated that the differential phenotype between *clpp3‐1* and *clpp5‐1* is in part due to the higher copy number of CLPP5 (three per complex) than CLPP3 (one per complex). Through site‐directed mutagenesis and complementation of null mutants, this study tests if the catalytic contribution of CLPP3 and CLPP5 can be inactivated (while keeping the structural contribution) without functional consequences for growth and development.

So far, several (candidate) chloroplast CLP substrates have been identified based on their direct interaction with the CLPS1 adaptor, including GLUTR (Nishimura et al., 2013), which was subsequently confirmed as a CLP substrate (Apitz et al., 2016). Other candidate CLPS1 substrates are four enzymes in the chloroplast shikimate pathway (Nishimura et al., 2013). Furthermore, systematic screening of protein stability of the thylakoid copper transporter, PAA2/HMA8 (P‐type ATPase of Arabidopsis2/Heavy‐metal‐associated8) in various plastid protease mutants identified the CLPPR core and CLPC1 as essential components to degrade PAA2 under copper replete conditions (Tapken, Kim, Nishimura, van Wijk, & Pilon, 2015). Additionally, DEOXYXYLULOSE 5‐PHOSHATE (DXS) in the chloroplast isoprenoid biosynthesis pathway and PHYTOENE SYNTHASE (PSY) involved in carotenoid biosynthesis were reported as a CLP substrates (Pulido et al., 2016; Welsch et al., 2018). Other candidate substrates have been suggested based on comparative proteome analysis of a range of *clp* mutants in Arabidopsis, rice and tobacco, but here it is hard to distinguish between direct and indirect effects – reviewed in (Nishimura, Kato, & Sakamoto, 2016) and (Moreno et al., 2018; Wu et al., 2016).

An alternative approach to identify CLP substrates, termed substrate trapping, has been reported for various bacterial and fungal CLP protease systems (Feng et al., 2013; Flynn, Neher, Kim, Sauer, & Baker, 2003; Trentini et al., 2016). In this approach, the catalytic activity of the CLP core is inactivated through site‐mutagenesis of the serine residue within the catalytic triad (Ser‐His‐Asp), resulting in accumulation of substrates within the CLP core central cavity and facilitating their identification by tandem mass spectrometry (MS/MS). This in vivo trapping strategy has been applied for the homo‐tetrameric CLP complexes in the gram‐negative bacterium *E. coli* (Flynn et al., 2003), the gram‐positive *Staphylococcus aureus* (Feng et al., 2013) and *Bacillus subtilis* (Trentini et al., 2016), as well as the fungus *Podospora anserine* (Fischer, Langer, & Osiewacz, 2015), but not yet in plants. It is important to note that the inactivation of the CLP system in these species does not greatly affect viability. The subunit complexity and the essential nature of the chloroplast CLP core makes an in vivo CLP protease trapping approach in plants more challenging than in bacteria and fungi. In this study we used this approach for the *Arabidopsis* chloroplast CLP protease generating in vivo tagged CLP core complexes containing inactive serine to alanine variants in either CLPP3 or CLPP5. MS/MS of affinity‐purified tagged CLP cores from the various transformants showed that we successfully obtained highly enriched STREPII‐tagged CLP cores with all catalytically active CLPP3 and CLPP5 copies and with catalytically inactive CLPP3 or CLPP5 copies. Other chloroplast proteins were co‐purified with the CLP cores and we evaluate and discuss their significance as candidate substrates in the context of bottlenecks in the chloroplast CLP adaptor‐chaperone‐protease system.

## 2 METHODS

### 2.1 Plant materials, cloning, transformation, genotyping, RT‐PCR

The T‐DNA insertion lines for *CLPP3* (At1g66670) and *CLPP5* (At1g02560) are SALK_065330 (*clpp3‐1*) and SALK_007708 (*clpp5‐1*), respectively, as described in (Kim et al., 2013). Heterozygous *clpp3‐1* and *clpp5‐1* were used for complementation. To generate transgene constructs, 3.5 kb of *CLPP3* and 3.7 kb of *CLPP5* genomic DNA including 1 kb upstream and 800 bp downstream from the coding region were cloned using gene‐specific primers (primer set 1 – see Supporting information Table S1). A 24‐nucleotide sequence, for the 8‐amino acid strepll tag (WSHPQFEK), was introduced into the transgene before the stop codon by PCR amplification (primer set 2 – see Supporting information Table S1). To create the *CLPP3S164A‐STREPll* and *CLPP5S193A‐STREPII* constructs, the catalytic serine of CLPP3 and CLPP5 was substituted by alanine using PCR amplification using primer set 3. All PCR products were sub‐cloned into pCR8/GW/TOPO^®^vector (Invitrogen) and binary vector pMDC123 using Gateway^®^ LR Clonase^®^ II enzyme mix (Invitrogen) and then introduced into the *Agrobacterium tumefaciens* GV3101 by electroporation. Agrobacterium‐mediated transformation by the flower dip method was according to (Clough & Bent, 1998). Crude plant genomic DNA was extracted from grinding frozen leaf tissues in the gDNA extraction buffer (0.2 M Tris‐HCl, pH 7.5, 0.25 M NaCl, 0.025 M EDTA, pH 8.0, 0.5% SDS) and precipitated by isopropanol. Genotyping was performed using *CLPP3/5‐*specific (primer set 4), transgene‐specific primers (primer set 5), and T‐DNA specific primers (primer set 6) Total RNA was extracted from grinding frozen leaf tissues using RNeasy Plant Mini Kit (Qiagen). The first‐strand of cDNA was synthesized using SuperScript^®^ III First‐Strand Synthesis System (Invitrogen) and then used for the synthesis of the second‐strand cDNA. Primers are listed in Supporting information Table S1.

### 2.2 Plant growth and phenotypic analysis

WT, *clpp3‐1* null, and complemented CLPP3‐STREPII and CLPP3S164A‐STREPII T2 seeds were surface disinfected with 70% ethanol for 15 min and then rinsed with 95% ethanol for three times before sowing on the half Murashige and Skoog (1/2 MS) plates. These plates were put in a cold room (4°C, dark) for 3 days stratification and then transferred into the growth chamber with 16/8 hr light/dark at 70 μmol photons m^−2^ s^−1^ for 12 days. Plates were relocated and observed every day. For soil‐grown phenotypic analysis, wt, complemented *CLPP3‐STREPII* and *CLPP3S164A‐STREPII* T3 seeds were sown on soil, put in a cold room for 3 days stratification, and then transferred in a growth chamber (16/8 hr light/dark at 120–150 μmol photons m^−2^ s^−1^). These soil‐grown plants were observed every 2 days. The plant height and the number of the rosette leaves were recorded for growth and development rates. Complemented CLPP5‐STREPII and transgenic CLPP5S193A‐STREPII were grown side by side on soil after 5 days stratification and then grown under 10/14 hr light/dark at 120–150 μmol photons m^−2^ s^−1^. BASTA‐spraying was performed every 2–3 days for the first 14 days. Surviving CLPP5‐STREPII and CLPP5S193A‐STREPII were genotyped and observed every week. At least ten developing siliques with similar size (length 1.8–2.0 cm) from heterozygous *clpp5‐1* and heterozygous *clpp5‐1* with *CLPP5S193A‐STREPII* T2 plants were examined under the dissecting microscope. Seeds were counted according to their colors.

### 2.3 SDS‐PAGE, BN‐PAGE, and immunoblot

Total leaf protein extraction under denaturing condition and non‐denaturing condition was performed according to (Friso, Olinares, & van Wijk, 2011) and (Olinares et al., 2011), respectively. Isolation of stromal proteins was according to (Kim et al., 2015). For immunoblot analysis of transgene expression and the assembly state of CLPPRT complexes, 20 μg of total leaf soluble proteins or 10–50 μg of stromal proteins were separated on SDS‐PAGE or Novex pre‐cast Bis‐Tris 4–16% gel (Invitrogen), transferred onto nitrocellulose or PVDF membranes, and stained with Ponceau S solution (0.3% Ponceau in 3% TCA). Immunoblot blotting using anti‐STREPII, anti‐CLPR2 and, anti‐CLPP3 antiserum was according to (Kim et al., 2015).

### 2.4 Affinity purification of STREPII‐tagged CLP complexes and MS/MS analysis

At least 2 mg stromal proteins or 24 mg total leaf proteins extracted under the non‐denaturing conditions was loaded on a self‐packed StrepTactin column using the superflow high capacity resin (IBA) according to (Olinares et al., 2011), except that 5 mM biotin instead of 2.5 mM desthiobiotin was included in the elution step. To minimize endogenous biotin‐conjugated proteins binding to the StrepTactin columns, avidin (0.2 mg per g leaf tissue) was included in the extraction buffer during total soluble protein extraction from CLPP5‐STREPII and CLPP5S193A‐STREPII lines. Amicon and Microcon spin concentrators (3 kDa) were used for concentration of the eluates before further analysis. Concentrated eluates were separated on 10.5–14% precast gels (Biorad), followed by the MS‐compatible silver stain. Comparative proteome analysis using a LTQ‐Orbitrap mass spectrometer, data processing, database searches, quantification of the relative protein abundance, as well as the selection of the best gene models followed the procedures described in Friso et al. (2011). We evaluated the samples for potential enrichment based on matched MS/MS adjusted spectra (adjSPC) normalized to the total number of adjSPC in each sample, resulting in NadjSPC. Annotations are from the Plant Proteome Data Base (http://ppdb.tc.cornell.edu/).

## 3 RESULTS AND DISCUSSION

### 3.1 Phenotypic analysis shows that the catalytic activity of CLPP3 is not needed for function

The catalytic triad Ser‐His‐Asp in CLPP proteases is widely conserved across bacterial CLPP proteins as well mitochondrial and plastid CLPP (but not in the catalytically inactive CLPR proteins) (Kim & Kim, 2008; Olinares et al., 2011; Peltier et al., 2004) (Supporting information Figure S1). Changing the serine residue into an alanine is sufficient to completely block proteolytic activity of CLPP proteases in all known cases, *e.g*. *E. coli* (Flynn, Levchenko, Sauer, & Baker, 2004)*, Mycobacterium tuberculosis* (Raju et al., 2012), *Bacillus subtilis* (Trentini et al., 2016) and *Synechococcus elongates* (Andersson et al., 2009).

**Figure 1:**
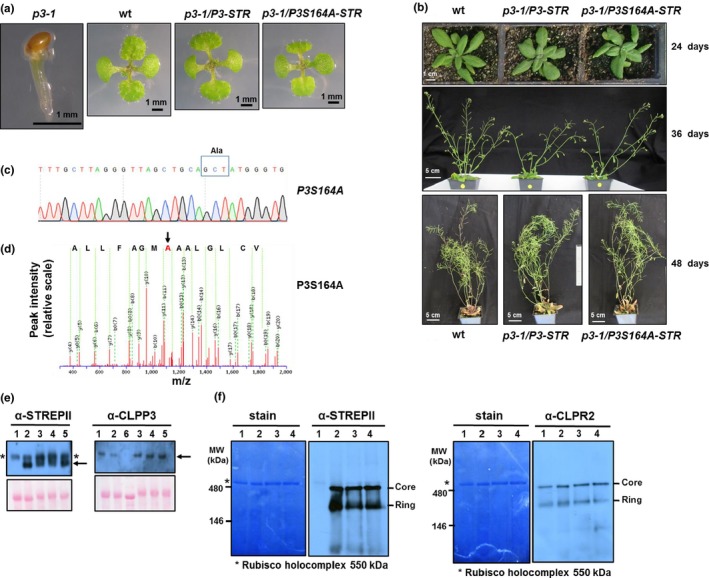
Characterization of *clpp3‐1* complemented with *CLPP3‐STREPll* and *CLPP3S164A‐STREPll*. (a) Seedlings of wt, *clpp3‐1* and *clpp3‐1* complemented with either *CLPP3*‐*STREPII* or *CLPP3S164A‐STREPII* grown on half MS medium (no sucrose) for 12 days under 16/8 hr light/dark cycle at 70 μmol photons m^−2^ s^−1^. Plants are homozygous for the STREPII transgenes. (b) Different developmental stages of soil‐grown wt and *clpp3‐1* complemented with either *CLPP3‐STREPII* or *CLPP3S164A‐STREPII*. Plants were grown on soil under 16/8 hr light/dark cycle at 120–150 μmol photons m^−2^ s^−1^. Plants are homozygous for the STREPII transgenes. (c) Confirmation of the point mutation in the catalytic site of *CLPP3S164A‐STREPII* by DNA sequencing of RT‐PCR products (primers #6 and #9) amplified from transgenic plants. The codon responsible for the serine to alanine point mutation is boxed and is TCT in wt but GCT in the mutant. (d) Confirmation of the point mutation S164 to A164 in the catalytic site of CLPP3S164A‐STREPII by MS/MS of the tryptic peptide (ADVSTVCLGLAAA_164_ MGAFLLASGSK) generated by tryptic digestion of affinity purified CLP complexes. The MS/MS spectrum is from a doubly charged precursor ion with *m*/*z* of 1155.5967 (2^+^) with MASCOT ion score of 131 (0.38 ppm error) and supports the residue A164. The partial peptide sequence listed above the spectrum (ALLFAGMA_164_AALGLCV) shown is based on y‐ions explaining the reverse order of amino acids. A list of b‐ and y‐ions is provided in Supporting information Figure S3A. An example of an MS/MS spectrum of the analogous CLPP3 wild‐type peptide is provided in Supporting information Figure S3B. (e) SDS‐PAGE gel and immunoblotting of total soluble protein from wt (lane 1), *clpp3‐1* with CLPP3**‐STREPll (lane 2), total soluble protein from *clpp3‐1* with *CLPP3‐STREPII* (lane 3) and *clpp3‐1* with *CLPP3S164A‐STREPII* (lanes 4 and 5), and stromal protein from *clpp3‐1* (lane 6). Arrows indicate CLPP3‐STREPll or CLPP3S164A‐STREPll proteins. *Indicates the nonspecific reaction. The Ponceau red stain of the blot illustrates protein loading. **35‐S driven cDNA of CLPP3‐STREPII as described in (Kim et al., 2013); all other lines and samples in the current study are using genomic CLP DNA for transgene expression (see Methods). Anti‐CLPP3 or anti‐STREPII serum was used. (f) Native gels and immunoblotting of stromal proteomes of wt (lane 1), *clpp3‐1* with CLPP3**‐STREPll (lane 2), *clpp3‐1* with CLPP3‐STREPll ((lane 3), and *clpp3‐1* with CLPP3S164A‐STREPll (lane 4). The intact CLPPR core and individual rings are indicated. *Indicates the Rubisco complex. Anti‐STREPII or anti‐CLPR2 serum was used for visualization of the core CLP complex and R‐ring. **35‐S driven cDNA of CLPP3‐strepll as described in (Kim et al., 2013)

To determine if the contribution of CLPP3 to the core complex is through its unique structure or also by contributing a catalytic site to the core complex, we transformed heterozygous *clpp3‐1* mutants with genomic *CLPP3* with a C‐terminal STREPII tag (*CLPP3‐STREPII*), or the same genomic construct but with a serine to alanine mutation in the catalytic site (*CLPP3S164A‐STREPII*) rendering CLPP3 catalytically inactive. Following growth on selective medium and genotyping of the T1 and T2 populations, we obtained multiple homozygous *clpp3‐1* lines expressing either *CLPP3‐STREPII* or *CLPP3S164A‐STREPII* (Figure 1a,b; Supporting information Figure S2A–C). No significant visible differences were observed among these complemented *clpp3‐1* lines and wt, as they all germinated and developed without the need for sucrose, as well as displayed normal green rosettes and similar growth and development (Figure 1a,b). In contrast, *clpp3‐1* nulls were arrested at the cotyledon stage (Figure 1a), as described previously (Kim et al., 2013).

The point mutation in CLPP3 was confirmed by DNA sequencing of RT‐PCR products generated by *CLPP3* and *STREPII* specific primers (Figure 1c). Furthermore, MS/MS of affinity purified CLP complexes identified the specific point mutation S164A in CLPP3 (Figure 1d; Supporting information Figure S3), thus further confirming that the point mutation in CLPP3 was successfully introduced in these lines. Accumulation of the transgenic CLPP3‐STREPII and CLPP3S164A‐STREPII proteins in vivo was also confirmed by immunoblotting of the denaturing soluble proteome separated by SDS‐PAGE and detected with anti‐STREPII and anti‐CLPP3 antisera (Figure 1e). To test if STREPII‐tagged CLPP3 and CLPP3S164A proteins normally assembled into CLPRT complexes, soluble leaf proteomes were separated by native gel electrophoresis (BN‐PAGE), followed by immunoblotting (Figure 1f). The similar size and migration pattern of CLPPRT complexes on BN‐PAGE for wt and complemented lines indicated that (i) these STREPII‐tagged transgenic proteins assembled into CLPPRT complexes in vivo*,* and (ii) the S‐to‐A change in CLPP3 and the STREPII tag did not interfere with the assembly state of CLPPRT complexes. Collectively, this shows that both catalytically active CLPP3‐STREPII and catalytic inactive CLPP3S164A‐STREPII successfully complement the developmental arrest of *clpp3‐1* null mutants. It can thus be concluded that CLPP3 makes a unique structural contribution to the CLPPR complex but that its catalytic activity is dispensable for plant growth and development.

### 3.2 The catalytic activity of CLPP5 is required for function; S‐to‐A change in CLPP5 prevents complementation of clpp5‐1

**Figure 2:**
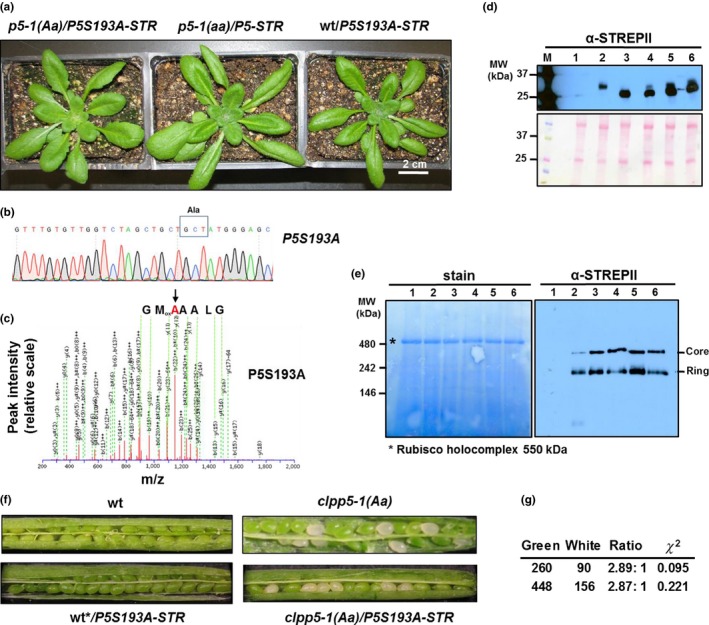
Characterization of wt and *clpp5‐1* lines expressing *CLPP5S193A‐STREPII* or *CLPP5‐STREPll* transgenes. (a) Phenotypic analysis of wt and heterozygous (Aa) *clpp5‐1* expressing *CLPP5S193A‐STREPll* or homozygous *clpp5‐1* expressing *CLPP5‐STREPII* grown on soil for 21 days under 10/14 hr light/dark cycle and then 11 days under 14/10 hr light/dark cycle all at 120–150 μmol photons m^−2^ s^−1^. (b) Confirmation of the point mutation in the catalytic sites of *CLPP5S193A‐STREPII* by DNA sequencing of RT‐PCR products (primers #14 and #9) amplified from transgenic plants. The codon responsible for the Ser to Ala point mutation is boxed and is AGT in wt but GCT in the mutant. (c) Confirmation of the point mutation in the catalytic sites of CLPP5S193A‐STREPII by MS/MS of the tryptic peptide (HIRPDVSTVCVGLAAA_193_ MGAFLLSAGTK) generated by tryptic digestion of affinity purified CLP complexes. The MS/MS spectrum is from a triply charged precursor ion with m/z of 914.8238 (3^+^) with MASCOT ion score of 83 (−5 ppm error) and supports the residue A193. The partial peptide sequence listed above the spectrum (GMA_193_AALGL) shown is based on y‐ions explaining this reads in reverse order. A list of b‐ and y‐ions is provided in Supporting information Figure S4A. An example of an MS/MS spectrum of the analogous CLPP5 wild‐type peptide is provided in Supporting information Figure S4B. (d) SDS‐PAGE and immunoblotting of total soluble protein from wt (lane 1), *clpp3‐1* with *CLPP3‐STREPIl* (lane 2), *clpp5‐1* with *CLPP5‐STREPII* (lane 3), and *clpp5‐1* heterozygous mutant with *CLPP5S193A‐STREPII* (lanes 4–6). The Ponceau red stain of the blot is shown. Anti‐STREPII serum was used. (e) Native gels and immunoblotting of total leaf protein from wt (lane 1), *clpp3‐1* with *CLPP3‐STREPII* (lane 2), *clpp5‐1* with *CLPP5‐STREPII* (lanes 3 and 5), the *clpp5‐1* heterozygous mutant with *CLPP5S193A‐STREPII* (lanes 4 and 6). The CLPPRT core and ring are indicated. Anti‐STREPII serum was used. *Indicates the Rubisco complex. (f) Developing siliques of wt, heterozygous *clpp5‐1 (Aa),* heterozygous *clpp5‐1 (Aa)* with *CLPP5S193A‐STREPll,* and wt* with *CLPP5S193A‐STREPII* obtained from a prior segregating progeny of heterozygous *clpp5‐1* with *CLPP5S193A‐STREPll*. The segregating white seeds are indicative of impaired chloroplast development due to the homozygous *clpp5‐1* background. (g) Segregation analysis of green and white seeds in developing siliques of two heterozygous *clpp5‐1* mutants expressing *CLPP5S193A‐STREPII*

Similar as for CLPP3, heterozygous *clpp5‐1* was transformed with genomic *CLPP5* with a C‐terminal STREPII tag (*CLPP5‐STREPII*), or the same construct but with a serine to alanine mutation in the catalytic site (*CLPP5S193A‐STREPII*) that rendered CLPP5 catalytically inactive. Expression of *CLPP5‐STREPII* could fully complement the *clpp5‐1* null mutant (Figure 2a – middle panel) but *CLPP5S193A‐STREPII* could not. We also identified several lines expressing *CLPP5S193A‐STREPII* in wt background or in the heterozygous *clpp5‐1* background (Supporting information Figure S2A,D); these lines did not show any visible phenotype (Figure 2a). The S193A mutation was confirmed by sequencing of RT‐PCR products, as well at the protein level by MS/MS‐based identification of peptides covering this catalytic residue (Figure 2b,c; Supporting information Figure S4). Furthermore, similar as we determined for CLPP3, STREPII‐tagged CLPP5 and CLPP5S193A accumulated at comparable levels (Figure 2d) and normally assembled into CLPPRT complexes (Figure 2e). The fact that *clpp5‐1(Aa)/CLPP5S193A‐STREPII* does not have a visible phenotype, indicates that accumulation of the catalytically inactive CLPP5 in presence of endogenous CLPP5 does not reduce the CLP protease capacity below the minimum threshold level required for chloroplast biogenesis, proteostasis, and function.

Despite extensive screening efforts, no homozygous *clpp5‐1* lines expressing *CLPP5S193A‐STREPII* were identified, in contrast to the many homozygous *clpp5‐1* lines expressing *CLPP5‐STREPII*. This suggests that complete loss of catalytic activity of CLPP5 results in embryo lethality. To further test this hypothesis, we analyzed the segregation pattern of the phenotypes of developing seeds in the siliques of wt, wt expressing *CLPP5S193A‐STREPII,* heterozygous *clpp5‐1*, and heterozygous *clpp5‐1* expressing *CLPP5S193A‐STREPII*. The first two lines produced only green seeds in developing siliques, while heterozygous *clpp5‐1* made both green and white seeds in a 3:1 ratio (Figure 2f), similar as previously observed (Kim et al., 2013). Importantly, ~3:1 segregating ratios were also found in the progeny of heterozygous *clpp5‐1* carrying *CLPP5S193A‐STREPII* (Figure 2f,g). This confirms that the serine to alanine change of CLPP5 prevented complementation of *clpp5‐1* null mutants, and thus that the complete loss of catalytic activity of CLPP5 which reduces the number of catalytic triads per complex from 10 to 7, results in embryo lethality.

### 3.3 Affinity‐purification of CLPP3 and CLPP5 STREPII‐tagged complexes

To identify potential substrates using the CLP core trapping technique as explained in the INTRODUCTION, we carried out replicate affinity experiments for homozygous *clpp3‐1* complemented with *CLPP3S164A‐STREPII* and using *clpp3‐1* complemented by *CLPP3‐STREPII* as a control. In the case of CLPP5, we used heterozygous *clpp5‐1* expressing *CLPP5S193A‐STREPII*, with homozygous *clpp5‐1* expressing *CLPP5‐STREPII* as the control. The STREPII‐tagged complexes were purified on streptactin columns. The affinity eluates were each run out on SDS‐PAGE gels, and each gel lane was cut into gel slices, followed by digestion with trypsin and MS/MS analysis for protein identification.

**Figure 3:**
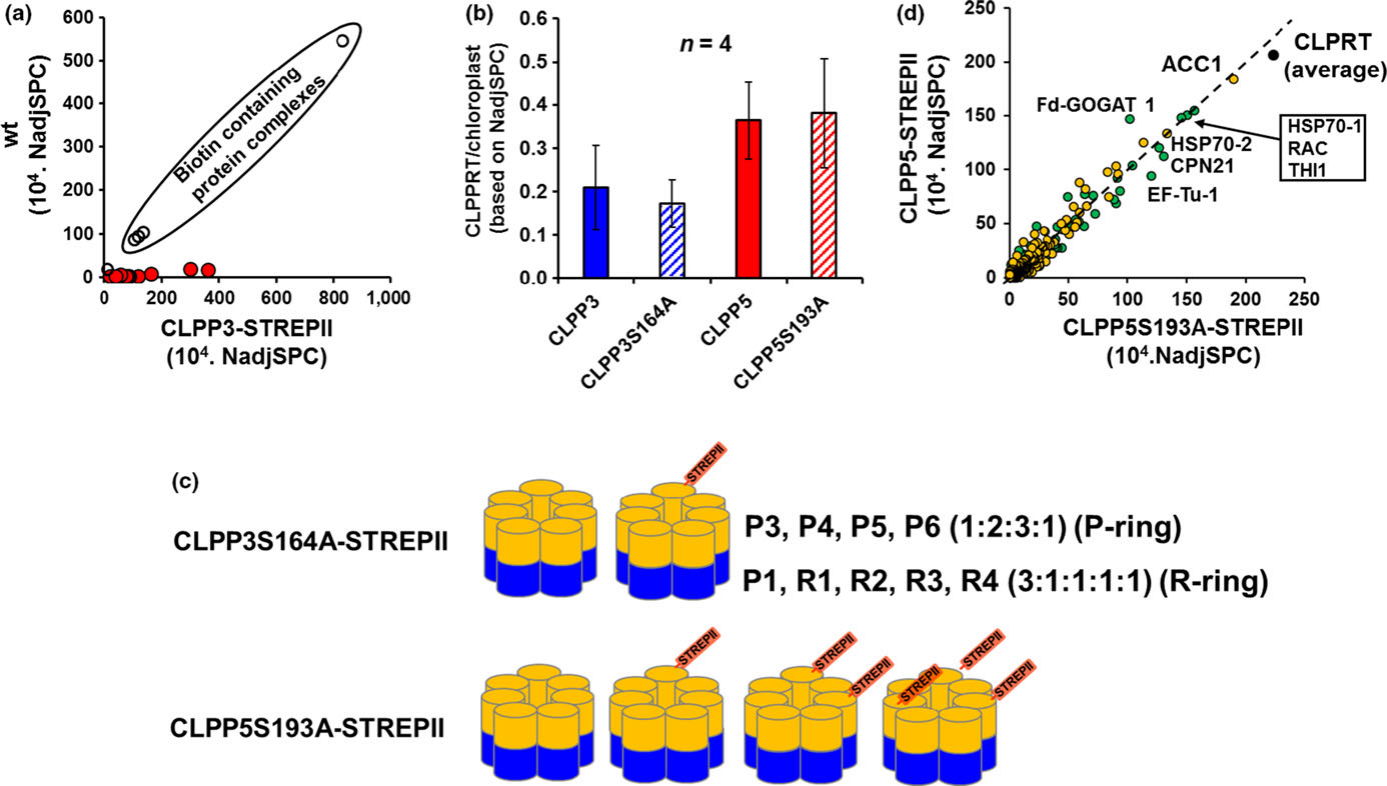
Affinity purification of STREPII‐tagged CLP core complexes and MS/MS analysis. (a) Relative abundance of endogenous Arabidopsis biotin‐binding ACC1, MCCA, BIOTIN CARBOXYLASE (BC) and MCCB, and subunits of the CLPPR complex in streptactin affinity eluates from wt (col‐o) plants and CLPP3‐STREPII transgenic lines. CLP subunits are marked in red‐filled circles. In these experiments, no avidin was added prior to affinity purifications; addition of avidin does reduce the binding of endogenous biotin protein proteins (see Supporting information Figure S5). (b) The average yield of CLPPRT subunits for affinity‐purified complexes containing CLPP3‐STREPII, CLPP3S164A‐STREPII, CLPP5‐STREPII and CLPP5S193A‐STREPII (*n* = 4 for each transgene). Yields were normalized to the total amount of detected plastid proteins, using NadjSPC as the basis for quantification. (c) Schematic representation of the wt and tagged CLPPR core complexes with P‐rings and R‐ring. Both ClpP3 and CLPP5 are part of the P‐ring, with respectively one or three copies per ring in native complexes (Olinares et al., 2011). In the case of the STREPII‐tagged CLPP3 and CLPP3S164A lines, each complex has one STREPII‐tagged CLPP3 protein and no endogenous CLPP3. However in case of *CLPP5S193A‐STREPII* lines, plants have both endogenous CLPP5 and CLPP5S193A‐STREPII; affinity purified CLPP5 complexes from these lines have one, two or three CLPP5S193A‐STREPII subunits per P‐ring. We note that the transgenes are genomic constructs with their endogenous promoters. The positions of the STREPII tagged proteins within the P‐ring are unknown. (d) Cross‐correlation of proteins of average abundance (based on NadjSPC) identified in affinity eluates of *CLPP5‐STREPII* and *CLPP5S193A‐STREPII* lines. These proteins were observed two or more times across the eight preparations (4 of each STREPII line). Proteins localized in the chloroplasts are indicated in green and proteins located outside the chloroplast or without annotated subcellular locations are indicated in yellow; subcellular location was based on annotation of manually curated experimental evidence collected from information in the public domain and in‐house data (from PPDB). The data point labeled ‘CLPPRT’ is the average of all 11 CLPP, CLPR and CLPT subunits. ACC1 (ACETYL‐COA‐CARBOXYLASE 1; AT1G36160) has biotin as the cofactor and has therefore high affinity to the streptactin column. HSP70‐1 (AT4G24280); HSP70‐2 (AT5G49910); RAC – RUBISCO ACTIVASE (AT2G39730); CPN21 (AT5G20720); EF‐TU – ELONGATION FACTOR TU (AT4G20360); THI1‐THIAMIN BIOSYNTHESIS 1 (AT5G54770)

First, we directly (*i.e*. eluates were run on the same SDS‐PAGE gel and processed in parallel) compared a negative control affinity purification using soluble protein extracts of wt plants (*i.e*. these lack STREPII‐tagged proteins) with CLPP3‐STREPII and CLPP3S164A‐STREPII purifications (Supporting information Table S2A). Biotin‐containing proteins bind to streptactin columns and Arabidopsis contains three major endogenous biotin‐binding complexes, namely cytosolic (ACC1) and plastidic acetyl‐CoA carboxylase complexes (ACCase), mitochondrial 3‐methylcrotonyl‐CoA carboxylase complexes (MCC) (Nikolau, Ohlrogge, & Wurtele, 2003). Since the concentration of these complexes should be similar in wt and CLPP3‐STREPII lines, they serve as internal controls. We detected high numbers of MS/MS spectra for these endogenous biotin binding proteins (ACC1, MCCA, MCCB, and BIOTIN CARBOXYLASE (BC) – part of the ACCase) (Figure 3a). Indeed, direct comparison between affinity eluates of wt and the two CLPP3‐STREPII lines showed very similar levels of these endogenous biotin‐containing complexes (average ratio 1.34) (Figure 3a). This provides an internal calibrant for evaluating proteins enriched in the affinity eluates. In contrast to these endogenous biotin binding complexes, CLP subunits were on average 25‐fold higher in the CLPP3‐STREPII lines than wt plants (Figure 3a), showing that the affinity purification of CLP complexes worked well.

A total of 16 successful independent affinity experiments for CLPP3 (four for each CLPP3‐STREPII and CLPPP3S164A‐STREPII) and CLPP5 (four for each CLPP5‐STREPII and CLPP5S193A‐STREPII) were carried out. We note that for all CLPP5 affinity purifications, we reduced the level of co‐purifying endogenous biotin binding proteins by preincubation of total soluble leaf extract with avidin; yet, there was still sufficient endogenous biotin binders for internal calibration (Supporting information Figure S5). The identified proteins, their annotation and number of matched MS/MS spectra (adjSPC – see Methods for explanation) for each experiment are assembled in Table S2B. All chloroplast CLPPRT core subunits including CLPP (P1, P3‐6), CLPR (R1‐4), as well as CLPT1,2 subunits were identified. These subunits of the CLP complex represented on average 19% of all adjSPC matched to plastid proteins in the case of CLPP3‐STREPII and CLPP3S164A‐STREPII, and on average 37% in the case of CLPP5‐STREPII and CLPP5S193A‐STREPII (Figure 3b). This shows that the serine to alanine mutation did not affect the yield of the CLPPR core complexes. We also identified low levels of CLPC1,2 chaperones in 14 out of 16 experiments without significant differences between the four STREPII tagged lines (Table S2B). We did not identify the adaptor CLPS1 and only once putative co‐adaptor CLPF (with 3 MS/MS spectra in a CLPP5S193A experiment). The lack of consistent observation of these adaptors is consistent with their substrate delivery role and transient interaction to CLPC chaperones (Nishimura et al., 2013, 2015). Together, these affinity experiments indicate that (i) the loss of catalytic triads in CLPP3 or CLPP5 did not affect the yield of CLP core affinity purification, complex composition or interaction between the CLPC and the CLP core, and that (ii) the CLPP5‐STREPII affinity purification was ~2‐fold more efficient than the CLPP3‐STREPII purification (Figure 3b), likely because there were on average more CLPP5‐STREPII copies per CLP core complex as illustrated in Figure 3c.

### 3.4 Trapping substrates in affinity‐purified CLPP3S164A‐STREPII and CLPP5S193A‐STREPII complexes

We then evaluated the affinity experiments for other (non‐CLP) chloroplast proteins enriched in these CLPP3‐ and CLPP5 STREPII‐tagged complexes; these could be interactors to the CLP core or CLP substrates located within the chamber of the protease core. The theoretical basis for trying to identify substrates in CLP cores is based on the hypothesis that chloroplast CLP complexes that lack one or more of the 10 catalytic sites (three in the R ring and seven in the P ring) have slower catalytic rates and therefore the average time to degrade substrates is extended; consequently such substrates reside for a longer time in the CLP core complex. MS/MS of such affinity enriched complexes could allow recognition of such substrates as having increased abundance when compared to fully active affinity‐enriched complexes. In the case of CLPP5, there are three endogenous copies per core (Olinares et al., 2011). Since we expressed the catalytically inactive CLPP5S193ASTREPII in the heterozygous *clpp5‐1* mutant, there are both endogenous CLPP5 subunits and inactive tagged CLPP5 copies. We note that the CLPP3 and CLPP5 transgenes are generated from genomic sequences including the endogenous promoters, as to ensure more natural levels of expression. The affinity purified STREPII‐tagged CLP cores must have either one, or possibly two or three inactive CLPP5 subunits (Figure 3c); in particular those complexes with 2 or 3 inactive CLPP5 subunits could have reduced CLP catalytic rates. However, since it is quite likely that the CLP protease is constantly involved in monitoring and degrading chloroplast proteins, it is possible that also fully active CLP core complexes contain substrates. Therefore, it is certainly possible that proteins observed in both unmodified CLPP3/5‐STREPII and CLPP3/5‐mutant‐STREPII could be substrates. However, for such proteins there are no good criteria to distinguish between proteins identified in the eluates due to unspecific protein interactions with e.g. the outer surface of the CLP core complex, and proteins residing within the chamber of the proteolytic CLP core because they were selected for degradation. One of the key objectives of the in vivo trapping approach is to obtain a strong candidate list of substrates, such that these can then be further investigated in the context of selection and degradation by the CLP protease system.

We compared the eluates of the wt and mutant forms of CLPP3‐ and CLPP5‐STREPII‐tagged complexes based on the normalized number of matched MS/MS spectra (NadjSPC) (Supporting information Table S2B). We tested two types of thresholds, based on ideas from other protease trapping studies (Arends, Thomanek, Kuhlmann, Marcus, & Narberhaus, 2016; Arends et al., 2018; Fischer et al., 2015; Trentini et al., 2016) and our own experience with protein affinity experiments, *e.g*. (Kim et al., 2015; Nishimura et al., 2013) to identify possible candidate substrates: (i) only observed in eluates of mutant complexes and not in CLPP3/5‐STREPII controls, or (ii) at least 2‐fold enriched in eluates of the mutant complexes compared to CLPP3/5‐STREPII controls. To reduce stochastic noise, we applied also either a minimum frequency of observation (2 or 3) across replicates or an abundance threshold of 1.10^−3^ based on NadjSPC (this corresponds to ~0.1% of protein mass).

In the case of CLPP3, we isolated chloroplasts and extracted soluble stromal proteomes and carried out two independent affinity purifications for each CLPP3‐STREPII and CLPP3S164A‐STREPII (trap) (Table S2B). 17 proteins were only found in the mutant and 12 proteins were >2× fold enriched in the mutant eluates; however because the number of matched MS/MS spectra were low and/or variable across replicates, we did not consider them strong candidate substrates. For comparison we note that the average ratio between CLP core subunits between CLPP3S164A‐STREPII and CLPP3‐STREPII was 0.7 and the ratio for stromal biotin carboxylase (BC) was 0.77. In a second set of CLPP3 experiments, we used soluble total leaf extracts and a total of four affinity purifications (two mutants and two controls). The relative average ratio of the yield CLP core subunits between CLPP3S164A‐STREPII and CLPP3‐STREPII was 1.0 and the ratio for endogenous biotin binder and internal control ACCase was 1.2. Applying these same thresholds (only in the mutant P3 or >2× enriched) we identified nine proteins that were more than 2‐fold enriched; however, following closer inspection we do not suggest them to be strong candidates, based on the same arguments as for the stromal replicates (Supporting information Table S2B).

In the case of CLPP5, our dataset consists of four affinity experiments with each CLPP5S193A‐STREPII and CLPP5‐STREPII and using total soluble leaf extracts (Supporting information Table S2B). Figure 3d shows a cross‐correlation of the average relative protein abundances (based on NadjSPC) across the replicate affinity purified CLPP5S193A‐STREPII and CLPP5‐STREPII complexes. This shows that the plastid and non‐plastid proteins co‐purified with the fully active and partially active complexes have generally similar relative abundances. Only one plastid protein was enriched, namely FRUCTOSE‐BISPHOSPHATASE (FBPA; HCEF1; AT3G54050) (Livingston, Cruz, Kohzuma, Dhingra, & Kramer, 2010) with an average ratio of 2.2 (standard deviation 1.1). Interesting, the abundant stromal proteins THI1 and EF‐TU were previously suggested to be CLP substrates (Moreno et al., 2018; Nishimura & van Wijk, 2015) and were observed with high abundance in the eluates of both CLPP5‐STREPII and CLPP5S193A‐STREPII (Figure 3d). However, they were also observed in the initial negative control using wt plants (*i.e*. they do not contain any STREPII‐tagged CLP proteins) (Supporting information Table S2A), rendering their presence in the eluates of the CLPP5‐STREPII and CLPP5S193A‐STREPII less significant. Furthermore stromal CPN21, abundant in eluates of both CLPP5 constructs (mutant/control ratio 1.2 ± 1.2) (Figure 3d), was previously identified as a strong interactor in CLP affinity purification using CLPT1‐STREPII and CLPT2‐STREPII Arabidopsis lines (Kim et al., 2015).

### 3.5 The bottle‐neck for degradation is likely substrate recognition and unfolding by CLP adaptors and chaperones, upstream of the CLP core

We showed that CLPP3 (one copy per CLP core) makes an essential structural contribution, but that its catalytic site is dispensable for plant growth and development, whereas the catalytic activity of CLPP5 (3 copies per CLP core) is essential. Based on the extensive affinity experiments described in this study, we conclude that the CLP core catalytic activity in the CLPP3S164A‐STREPII and CLPP5 S193A‐STREPII complexes with reduced number of catalytic triads did not result in significant accumulation of substrates as compared to fully active CLP complexes. This suggests that the bottle‐neck for degradation is likely substrate recognition and unfolding by CLP adaptors and chaperones, upstream of the CLP core. In vivo substrate trapping through partial inactivation of unfolding activity of the CLPC/D chaperones could provide an alternative strategy for identification of candidate substrates. This has been a successful approach for the CLPC homolog in the gram‐positive bacterium *Staphylococcus aureus* where the CLP system is not essential for viability (Graham, Lei, & Lee, 2013).

## AUTHOR CONTRIBUTIONS

J.R.L. and K.J. V.W. designed the experiments, analyzed the data, and wrote the manuscript. J.R.L. and J.K. performed site‐mutagenesis. J.R.L conducted plant and molecular experiments. J.R.L prepared protein samples; G.F. performed all MS analysis. K.J.v.W obtained funding and provided oversight of the project.
