# Supplementary material for: Consequences of the loss of catalytic triads in chloroplast CLPPR protease core complexes in vivo
Source: Plant Direct. 2018 Oct 25;2(10):e00086. doi: 10.1002/pld3.86 (PMC6508832; doi:10.1002/pld3.86)
Supplement: Supplementary file 1 [file PLD3-2-e00086-s001.pdf]

```
HpClpP          ----------------------------MMGYIPYVI----ENTDRG--ERSYDIYSRLL     26
EcClpP          ------------------------------ALVPMVI----EQTSRG--ERSFDIYSRLL     24
SpClpP          -----------MGSSHHHHHHSSGLVPRGSHMIPVVI----EQTSRG--ERSYDIYSRLL     43
PfClpP          ------------------MGSSHHHHHHSSGRENLYFQGHM-----DIKDMKKDVKLFFF     37
MtClpP          ----------------------------MSQVTDMR-----SNSQGL--SLTDSVYERLL     25
ClpP3-cTP       ------------QTLSSNWDVSSF-------SIDSVA----QSPSRLPSFEELDTTNMLL     37
ClpP5-cTP       AVYSGNLWTPEIPSPQGVWSIRDDLQVPSSPYFPAYAQGQGPPPMVQ--ERFQSIISQLF     58
                                                             .    ::

HpClpP          KDRIVLLSGEINDSVASSIVAQLLFLEAEDPEKDIGLYINSPGGVITSGLSIYDTMNFIR     86
EcClpP          KERVIFLTGQVEDHMANLIVAQMLFLEAENPEKDIYLYINSPGGVITAGMSIYDTMQFIK     84
SpClpP          KDRIIMLTGPVEDNMANSVIAQLLFLDAQDSTKDIYLYVNTPGGSVSAGLAIVDTMNFIK     103
PfClpP          KKRIIYLTDEINKKTADELISQLLYLDNIN-HNDIKIYINSPGGSINEGLAILDIFNYIK     96
MtClpP          SERIIFLGSEVNDEIANRLCAQILLLAAEDASKDISLYINSPGGSISAGMAIYDTMVLAP     85
ClpP3-cTP       RQRIVFLGSQVDDMTADLVISQLLLLDAEDSERDITLFINSPGGSITAGMGIYDAMKQCK     97
ClpP5-cTP       QYRIIRCGGAVDDDMANIIVAQLLYLDAVDPTKDIVMYVNSPGGSVTAGMAIFDTMRHIR     118
                  *::   . ::.  *. : :*:* *   :  .** :::*:*** :. *:.* * :

HpClpP          PDVSTICIGQAASMGAFLLSCGAKGKRFSLPHSRIMIHQPLGGAQGQ--ASDIEIISNEI     144
EcClpP          PDVSTICMGQAASMGAFLLTAGAKGKRFCLPNSRVMIHQPLGGYQGQ--ATDIEIHAREI     142
SpClpP          ADVQTIVMGMAASMGTVIASSGAKGKRFMLPNAEYMIHQPMGGTGGGTQQTDMAIAPEHL     163
PfClpP          SDIQTISFGLVASMASVILASGKKGKRKSLPNCRIMIHQPLGNAFGH--PQDIEIQTKEI     154
MtClpP          CDIATYAMGMAASMGEFLLAAGTKGKRYALPHARILMHQPLGGVTGS--AADIAIQAEQF     143
ClpP3-cTP       ADVSTVCLGLAASMGAFLLASGSKGKRYCMPNSKVMIHQPLGTAGGK--ATEMSIRIREM     155
ClpP5-cTP       PDVSTVCVGLAASMGAFLLSAGTKGKRYSLPNSRIMIHQPLGGAQGG--QTDIDIQANEM     176
                 *: *  .* .***. .: :.* ****  :*:.. ::***:*   *     :: *  ..:

HpClpP          LRLKGLMNSILAQNSGQSLEQIAKDTDRDFYMSAKEAKEYGLIDKVLQKNVK--------     196
EcClpP          LKVKGRMNELMALHTGQSLEQIERDTERDRFLSAPEAVEYGLVDSILTHRN---------     193
SpClpP          LKTRNTLEKILAENSGQSMEKVHADAERDNWMSAQETLEYGFIDEIMANNSLNGS-----     218
PfClpP          LYLKKLLYHYLSSFTNQTVETIEKDSDRDYYMNALEAKQYGIIDEVIETKLPHPYFNKVE     214
MtClpP          AVIKKEMFRLNAEFTGQPIERIEADSDRDRWFTAAEALEYGFVDHIITRAHVNGEAQLEH     203
ClpP3-cTP       MYHKIKLNKIFSRITGKPESEIESDTDRDNFLNPWEAKEYGLIDAVIDDGKPGLIAPIGD     215
ClpP5-cTP       LHHKANLNGYLAYHTGQSLEKINQDTDRDFFMSAKEAKEYGLIDGVIMNPLKALQPLAAA     236
                   :  :    :  :.:  . :  *::** ::.  *: :**::* ::

HpClpP          ---------------------------------------- 196
EcClpP          ---------------------------------------- 193
SpClpP          ---------------------------------------- 218
PfClpP          K--------------------------------------- 215
MtClpP          HHHHH----------------------------------- 208
ClpP3-cTP       GTPPPKTKVWDLWKVEGTKKDNTNLPSERSMTQNGYAAIE 255
ClpP5-cTP       ---------------------------------------- 236
```

Supplemental Figure 1. Sequence alignment of CLPP3 and CLPP5 with CLPP homologs.

Crystalized CLPP homologs from *Helicobacter pylori* (hp), *Escherichia coli* (Ec), *Streptococcus pnneumoniae* (Sp), *Plasmodium falciparum* (Pf), and *Mycobacterium tuberculosis* (Mt) are aligned and compared with CLPP3 and CLPP5 without cTP. The residues in the catalytic triad are indicated with arrowheads. sequence identity (*); high sequence similarity (:); low level of similarity (.)

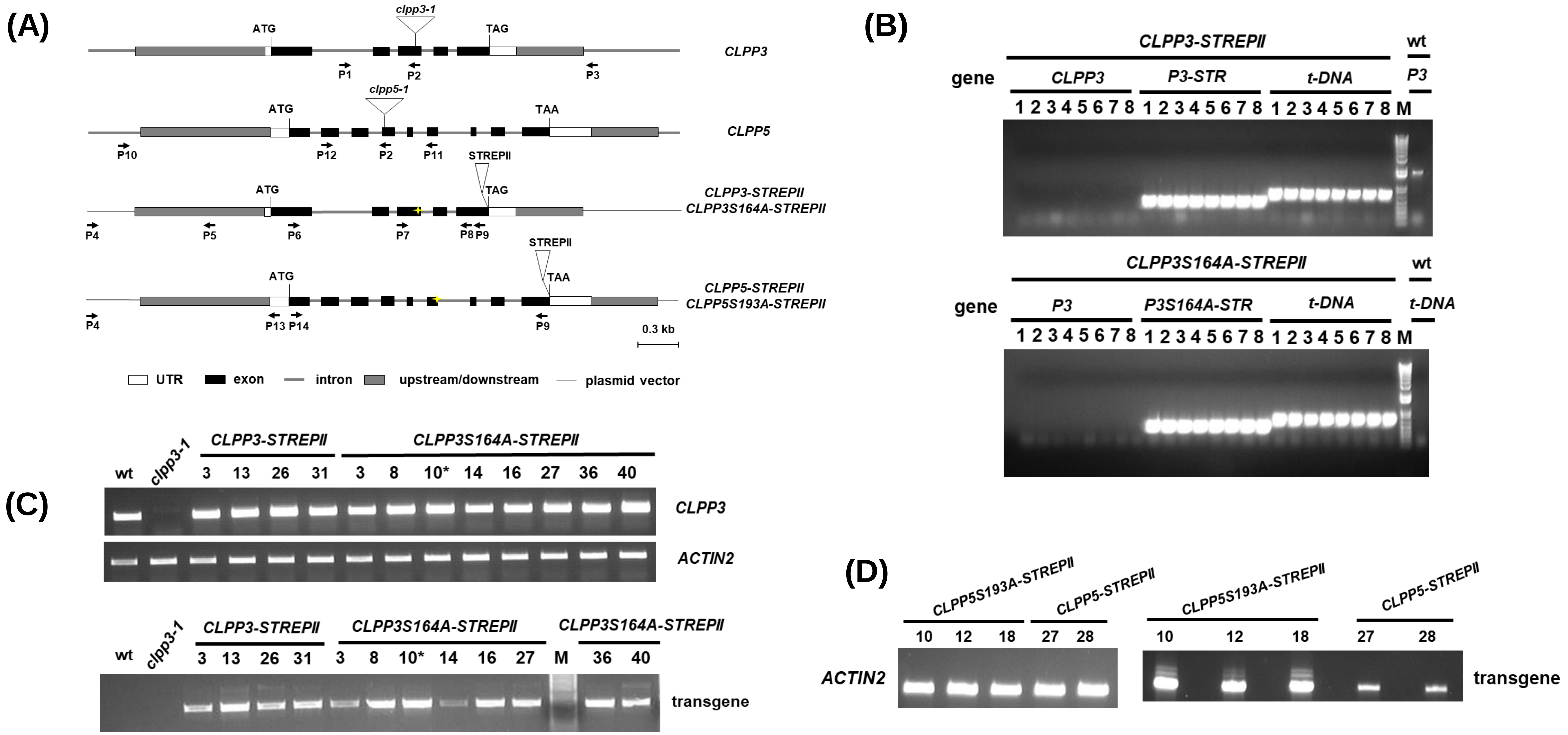

Supplemental Figure 2. Genotyping and RT-PCR analysis of transgenic lines.

(A) Gene models and the transgenic constructs for complementation. Primers used for genotyping and RT-PCR analysis are numbered (see primer # in Supplemental Table 1). The yellow star highlights the Ser-to-Ala change. The upstream/downstream region and the plastid vector shown are not to scale.

(B) Genotyping for endogenous *CLPP3* (P3; primers P1+P3), transgenic *CLPP3-STREPII* or *CLPP3S164A-STREPII* (P3-STR; primers P4+P5), or the *t-DNA* in *clpp3-1* (primers P1+P2). Eight individual T2 plants from one *CLPP3-STREPII* T1 line in the *clpp3-1* background (upper panel) or *CLPP3S164A-STREPII* T1 line in the *clpp3-1* background (lower panel) were analyzed. wt plants (wt) was used as positive control for *CLPP3* and as the negative control for the *t-DNA*.

(C) RT-PCR analysis of endogenous *CLPP3* and *ACTIN2* (upper panel) and *CLPP3-STREPII* or *CLPP3S164A-STREPll* expression (primer pairs P7+P8 or P6+P9) in the transgenic T1 lines. Four putative *CLPP3-STREPII* T1 or eight putative *CLPP3S164A-STREPll* T1 plants were analyzed. * - removed from further analysis. wt and *clpp3-1* lines were used as control plants.

(D) RT-PCR analysis of *CLPP5-STREPII* or *CLPP5S193A-STREPll* expression (P14+P9) in the two putative *CLPP5-STREPII* and three putative *CLPP5S193A-STREPII* T1 lines. *ACTIN2* was used as an internal control.

(A)

CLPP3S164A-STREPII

Precursor Ion = 1155.5967 (2^+)
Mascot Score = 131
Measured Mass = 2309.1768
Calculated Mass = 2309.1759
ppm = 0.38

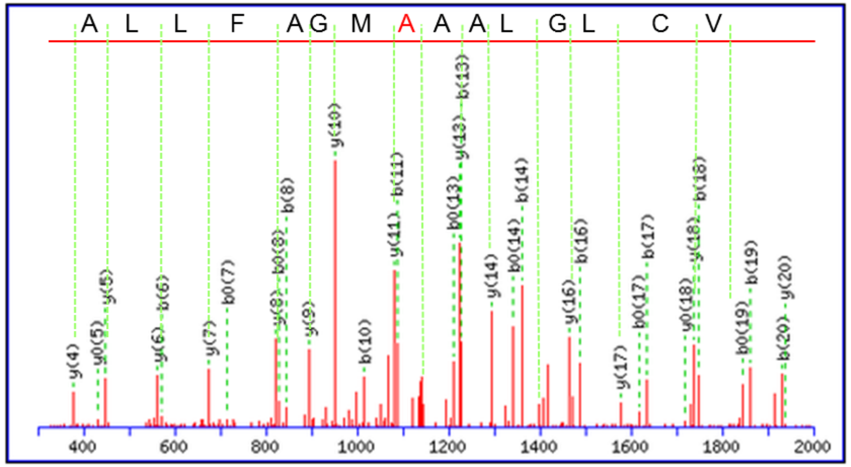

ADVSTVCLGLAAA$_{164}$MGAFLLASGSK

#	b	b^{++}	b^{0}	b^{0++}	Seq.	y	y^{++}	y^{*}	y^{*++}	y^{0}	y^{0++}	#
1	72.0444	36.5258			A							24
2	187.0713	94.0393	169.0608	85.0340	D	2239.1461	1120.0767	2222.1196	1111.5634	2221.1355	1111.0714	23
3	286.1397	143.5735	268.1292	134.5682	V	2124.1192	1062.5632	2107.0926	1054.0499	2106.1086	1053.5579	22
4	373.1718	187.0895	355.1612	178.0842	S	2025.0507	1013.0290	2008.0242	1004.5157	2007.0402	1004.0237	21
5	474.2195	237.6134	456.2089	228.6081	T	1938.0187	969.5130	1920.9922	960.9997	1920.0082	960.5077	20
6	573.2879	287.1476	555.2773	278.1423	V	1836.9710	918.9892	1819.9445	910.4759	1818.9605	909.9839	19
7	733.3185	367.1629	715.3080	358.1576	C	1737.9026	869.4550	1720.8761	860.9417	1719.8921	860.4497	18
8	846.4026	423.7049	828.3920	414.6996	L	1577.8720	789.4396	1560.8454	780.9264	1559.8614	780.4343	17
9	903.4240	452.2157	885.4135	443.2104	G	1464.7879	732.8976	1447.7614	724.3843	1446.7773	723.8923	16
10	1016.5081	508.7577	998.4975	499.7524	L	1407.7664	704.3869	1390.7399	695.8736	1389.7559	695.3816	15
11	1087.5452	544.2762	1069.5347	535.2710	A	1294.6824	647.8448	1277.6558	639.3316	1276.6718	638.8395	14
12	1158.5823	579.7948	1140.5718	570.7895	A	1223.6453	612.3263	1206.6187	603.8130	1205.6347	603.3210	13
13	1229.6195	615.3134	1211.6089	606.3081	A	1152.6082	576.8077	1135.5816	568.2944	1134.5976	567.8024	12
14	1360.6599	680.8336	1342.6494	671.8283	M	1081.5710	541.2892	1064.5445	532.7759	1063.5605	532.2839	11
15	1417.6814	709.3443	1399.6708	700.3391	G	950.5306	475.7689	933.5040	467.2556	932.5200	466.7636	10
16	1488.7185	744.8629	1470.7079	735.8576	A	893.5091	447.2582	876.4825	438.7449	875.4985	438.2529	9
17	1635.7869	818.3971	1617.7764	809.3918	F	822.4720	411.7396	805.4454	403.2264	804.4614	402.7343	8
18	1748.8710	874.9391	1730.8604	865.9339	L	675.4036	338.2054	658.3770	329.6921	657.3930	329.2001	7
19	1861.9551	931.4812	1843.9445	922.4759	L	562.3195	281.6634	545.2930	273.1501	544.3089	272.6581	6
20	1932.9922	966.9997	1914.9816	957.9944	A	449.2354	225.1214	432.2089	216.6081	431.2249	216.1161	5
21	2020.0242	1010.5157	2002.0136	1001.5105	S	378.1983	189.6028	361.1718	181.0895	360.1878	180.5975	4
22	2077.0457	1039.0265	2059.0351	1030.0212	G	291.1663	146.0868	274.1397	137.5735	273.1557	137.0815	3
23	2164.0777	1082.5425	2146.0671	1073.5372	S	234.1448	117.5761	217.1183	109.0628	216.1343	108.5708	2
24					K	147.1128	74.0600	130.0863	65.5468			1

(B)

CLPP3-STREPII

Precursor Ion = 1163.5893 (2^+)
Mascot Score = 131
Measured Mass = 2325.1641
Calculated Mass = 2325.1709
ppm = - 2.92

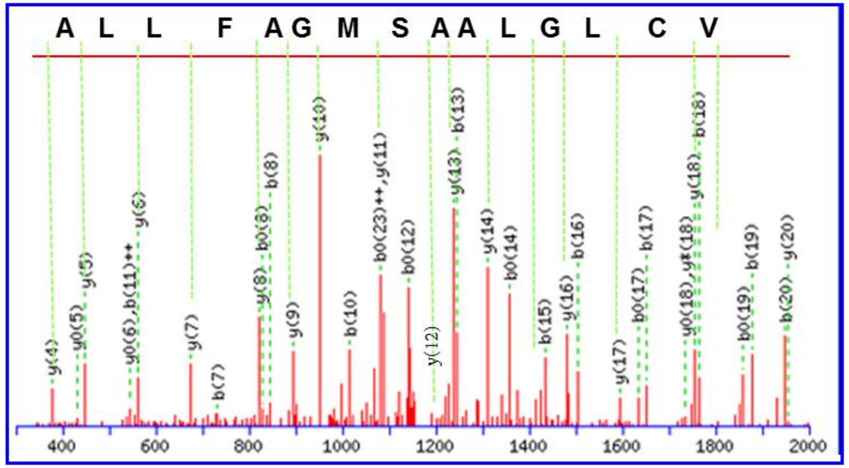

m/z

ADVSTVCLGLAAS$_{164}$MGAFLLASGSK

#	b	b^{++}	b^{0}	b^{0++}	Seq.	y	y^{++}	y^{*}	y^{*++}	y^{0}	y^{0++}	#
1	72.0444	36.5258			A							24
2	187.0713	94.0393	169.0608	85.0340	D	2255.1410	1128.0741	2238.1145	1119.5609	2237.1305	1119.0689	23
3	286.1397	143.5735	268.1292	134.5682	V	2140.1141	1070.5607	2123.0875	1062.0474	2122.1035	1061.5554	22
4	373.1718	187.0895	355.1612	178.0842	S	2041.0457	1021.0265	2024.0191	1012.5132	2023.0351	1012.0212	21
5	474.2195	237.6134	456.2089	228.6081	T	1954.0136	977.5105	1936.9871	968.9972	1936.0031	968.5052	20
6	573.2879	287.1476	555.2773	278.1423	V	1852.9660	926.9866	1835.9394	918.4733	1834.9554	917.9813	19
7	733.3185	367.1629	715.3080	358.1576	C	1753.8975	877.4524	1736.8710	868.9391	1735.8870	868.4471	18
8	846.4026	423.7049	828.3920	414.6996	L	1593.8669	797.4371	1576.8403	788.9238	1575.8563	788.4318	17
9	903.4240	452.2157	885.4135	443.2104	G	1480.7828	740.8951	1463.7563	732.3818	1462.7723	731.8898	16
10	1016.5081	508.7577	998.4975	499.7524	L	1423.7614	712.3843	1406.7348	703.8710	1405.7508	703.3790	15
11	1087.5452	544.2762	1069.5347	535.2710	A	1310.6773	655.8423	1293.6508	647.3290	1292.6667	646.8370	14
12	1158.5823	579.7948	1140.5718	570.7895	A	1239.6402	620.3237	1222.6136	611.8105	1221.6296	611.3184	13
13	1245.6144	623.3108	1227.6038	614.3055	S	1168.6031	584.8052	1151.5765	576.2919	1150.5925	575.7999	12
14	1376.6549	688.8311	1358.6443	679.8258	M	1081.5710	541.2892	1064.5445	532.7759	1063.5605	532.2839	11
15	1433.6763	717.3418	1415.6657	708.3365	G	950.5306	475.7689	933.5040	467.2556	932.5200	466.7636	10
16	1504.7134	752.8604	1486.7029	743.8551	A	893.5091	447.2582	876.4825	438.7449	875.4985	438.2529	9
17	1651.7818	826.3946	1633.7713	817.3893	F	822.4720	411.7396	805.4454	403.2264	804.4614	402.7343	8
18	1764.8659	882.9366	1746.8553	873.9313	L	675.4036	338.2054	658.3770	329.6921	657.3930	329.2001	7
19	1877.9500	939.4786	1859.9394	930.4733	L	562.3195	281.6634	545.2930	273.1501	544.3089	272.6581	6
20	1948.9871	974.9972	1930.9765	965.9919	A	449.2354	225.1214	432.2089	216.6081	431.2249	216.1161	5
21	2036.0191	1018.5132	2018.0085	1009.5079	S	378.1983	189.6028	361.1718	181.0895	360.1878	180.5975	4
22	2093.0406	1047.0239	2075.0300	1038.0186	G	291.1663	146.0868	274.1397	137.5735	273.1557	137.0815	3
23	2180.0726	1090.5399	2162.0620	1081.5347	S	234.1448	117.5761	217.1183	109.0628	216.1343	108.5708	2
24					K	147.1128	74.0600	130.0863	65.5468			1

Supplemental Figure 3. MS/MS-based verification of the S164A mutation in CLPP3S164A-STREPII and comparison to CLPP3-STREPII
(A) Identification of the point mutation S164 to A164 in the catalytic site of CLPP3S164A-STREPII by MS/MS of the tryptic peptide (ADVSTVCLGLAAAMGAFLLASGSK) generated by tryptic digestion of affinity purified CLP complexes. The MS/MS spectrum is from a doubly charged precursor ion with m/z of 1155.5967 (2^+) with MASCOT ion score of 131 (0.38 ppm error) and supports the residue A164. The partial peptide sequence listed above the spectrum (ALLFAGMA$_{164}$AALGLCV) shown is based on y-ions explaining the reverse order of amino acids. A full list of b- and y-ions is listed. This is the same spectrum as shown in Figure 1D.
(B) An example of an MS/MS spectrum of the CLPP3 wild-type peptide covering the region around S164. MS/MS of the tryptic peptide (ADVSTVCLGLAASMGAFLLASGSK) generated by tryptic digestion of affinity purified CLP complexes. The MS/MS spectrum is from a doubly charged precursor ion with m/z of 1163.5893 (2^+) with MASCOT ion score of 131 (-2.92 ppm error) and supports the residue S164. The partial peptide sequence listed above the spectrum (ALLFAGMS$_{164}$AALGLCV) shown is based on y-ions explaining this reads in reverse order. A full list of b- and y-ions is listed.

(A)

CLPP5-S193A-STREPII

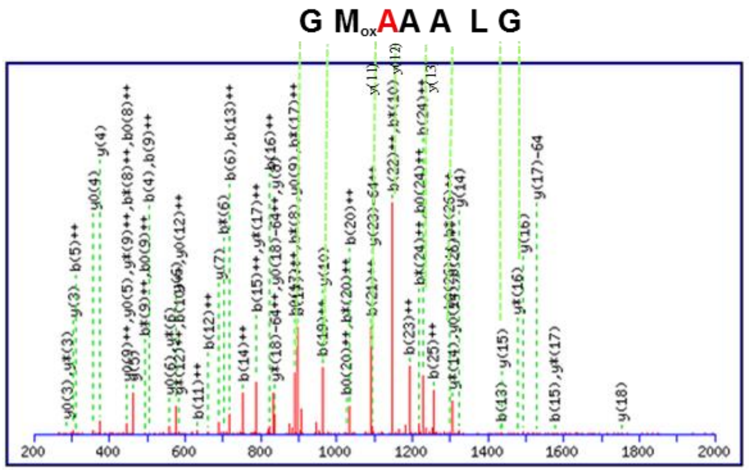

Precursor Ion = 914.8238 (3^+)
Mascot Score = 83
Measured Mass = 2741.43575
Calculated Mass = 2741.4495
ppm = -5.0

HIRPDVSTVCVGLAAA_{193}MGAFLLSAGTK

#	b	b^{++}	b^{*}	b^{*++}	b^{0}	b^{0++}	Seq.	y	y^{++}	y^{*}	y^{*++}	y^{0}	y^{0++}	#
1	138.0662	69.5367					H							27
2	251.1503	126.0788					I	2621.3790	1311.1931	2604.3524	1302.6798	2603.3684	1302.1878	26
3	407.2514	204.1293	390.2248	195.6160			R	2508.2949	1254.6511	2491.2683	1246.1378	2490.2843	1245.6458	25
4	504.3041	252.6557	487.2776	244.1424			P	2352.1938	1176.6005	2335.1672	1168.0873	2334.1832	1167.5952	24
5	619.3311	310.1692	602.3045	301.6559	601.3205	301.1639	D	2255.1410	1128.0741	2238.1145	1119.5609	2237.1305	1119.0689	23
6	718.3995	359.7034	701.3729	351.1901	700.3889	350.6981	V	2140.1141	1070.5607	2123.0875	1062.0474	2122.1035	1061.5554	22
7	805.4315	403.2194	788.4050	394.7061	787.4209	394.2141	S	2041.0457	1021.0265	2024.0191	1012.5132	2023.0351	1012.0212	21
8	906.4792	453.7432	889.4526	445.2300	888.4686	444.7380	T	1954.0136	977.5105	1936.9871	968.9972	1936.0031	968.5052	20
9	1005.5476	503.2774	988.5211	494.7642	987.5370	494.2722	V	1852.9660	926.9866	1835.9394	918.4733	1834.9554	917.9813	19
10	1165.5783	583.2928	1148.5517	574.7795	1147.5677	574.2875	C	1753.8975	877.4524	1736.8710	868.9391	1735.8870	868.4471	18
11	1264.6467	632.8270	1247.6201	624.3137	1246.6361	623.8217	V	1593.8669	797.4371	1576.8403	788.9238	1575.8563	788.4318	17
12	1321.6681	661.3377	1304.6416	652.8244	1303.6576	652.3324	G	1494.7985	747.9029	1477.7719	739.3896	1476.7879	738.8976	16
13	1434.7522	717.8797	1417.7256	709.3665	1416.7416	708.8745	L	1437.7770	719.3921	1420.7505	710.8789	1419.7665	710.3869	15
14	1505.7893	753.3983	1488.7628	744.8850	1487.7787	744.3930	A	1324.6930	662.8501	1307.6664	654.3368	1306.6824	653.8448	14
15	1576.8264	788.9168	1559.7999	780.4036	1558.8159	779.9116	A	1253.6558	627.3316	1236.6293	618.8183	1235.6453	618.3263	13
16	1647.8635	824.4354	1630.8370	815.9221	1629.8530	815.4301	A	1182.6187	591.8130	1165.5922	583.2997	1164.6082	582.8077	12
17	1794.8989	897.9531	1777.8724	889.4398	1776.8884	888.9478	M	1111.5816	556.2944	1094.5551	547.7812	1093.5710	547.2892	11
18	1851.9204	926.4638	1834.8939	917.9506	1833.9098	917.4586	G	964.5462	482.7767	947.5197	474.2635	946.5356	473.7715	10
19	1922.9575	961.9824	1905.9310	953.4691	1904.9470	952.9771	A	907.5247	454.2660	890.4982	445.7527	889.5142	445.2607	9
20	2070.0259	1035.5166	2052.9994	1027.0033	2052.0154	1026.5113	F	836.4876	418.7475	819.4611	410.2342	818.4771	409.7422	8
21	2183.1100	1092.0586	2166.0834	1083.5454	2165.0994	1083.0534	L	689.4192	345.2132	672.3927	336.7000	671.4087	336.2080	7
22	2296.1941	1148.6007	2279.1675	1140.0874	2278.1835	1139.5954	L	576.3352	288.6712	559.3086	280.1579	558.3246	279.6659	6
23	2383.2261	1192.1167	2366.1995	1183.6034	2365.2155	1183.1114	S	463.2511	232.1292	446.2245	223.6159	445.2405	223.1239	5
24	2454.2632	1227.6352	2437.2367	1219.1220	2436.2526	1218.6300	A	376.2191	188.6132	359.1925	180.0999	358.2085	179.6079	4
25	2511.2847	1256.1460	2494.2581	1247.6327	2493.2741	1247.1407	G	305.1819	153.0946	288.1554	144.5813	287.1714	144.0893	3
26	2612.3323	1306.6698	2595.3058	1298.1565	2594.3218	1297.6645	T	248.1605	124.5839	231.1339	116.0706	230.1499	115.5786	2
27							K	147.1128	74.0600	130.0863	65.5468			1

(B)

CLPP5-STREPII

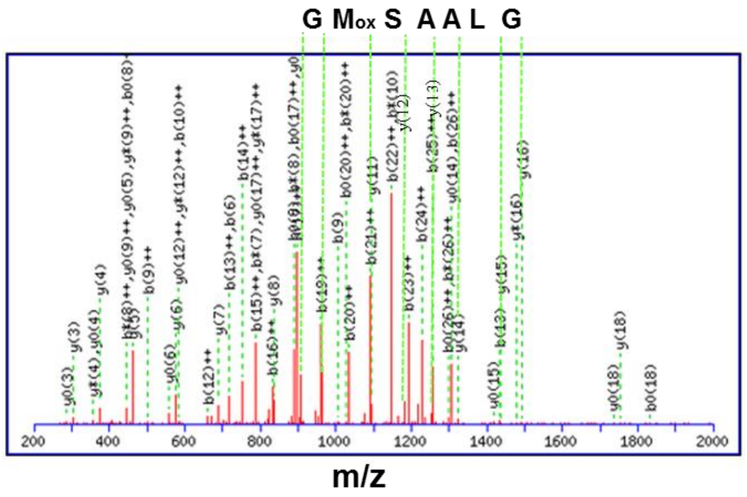

Precursor Ion = 920.150193 (3^+)
Mascot Score = 126
Measured Mass = 2757.428751
Calculated Mass = 2757.4306
ppm = 0.68

HIRPDVSTVCVGLAAS_{193}MGAFLLSAGTK

#	b	b^{++}	b^{*}	b^{*++}	b^{0}	b^{0++}	Seq.	y	y^{++}	y^{*}	y^{*++}	y^{0}	y^{0++}	#
1	138.0662	69.5367					H							27
2	251.1503	126.0788					I	2621.3790	1311.1931	2604.3524	1302.6798	2603.3684	1302.1878	26
3	407.2514	204.1293	390.2248	195.6160			R	2508.2949	1254.6511	2491.2683	1246.1378	2490.2843	1245.6458	25
4	504.3041	252.6557	487.2776	244.1424			P	2352.1938	1176.6005	2335.1672	1168.0873	2334.1832	1167.5952	24
5	619.3311	310.1692	602.3045	301.6559	601.3205	301.1639	D	2255.1410	1128.0741	2238.1145	1119.5609	2237.1305	1119.0689	23
6	718.3995	359.7034	701.3729	351.1901	700.3889	350.6981	V	2140.1141	1070.5607	2123.0875	1062.0474	2122.1035	1061.5554	22
7	805.4315	403.2194	788.4050	394.7061	787.4209	394.2141	S	2041.0457	1021.0265	2024.0191	1012.5132	2023.0351	1012.0212	21
8	906.4792	453.7432	889.4526	445.2300	888.4686	444.7380	T	1954.0136	977.5105	1936.9871	968.9972	1936.0031	968.5052	20
9	1005.5476	503.2774	988.5211	494.7642	987.5370	494.2722	V	1852.9660	926.9866	1835.9394	918.4733	1834.9554	917.9813	19
10	1165.5783	583.2928	1148.5517	574.7795	1147.5677	574.2875	C	1753.8975	877.4524	1736.8710	868.9391	1735.8870	868.4471	18
11	1264.6467	632.8270	1247.6201	624.3137	1246.6361	623.8217	V	1593.8669	797.4371	1576.8403	788.9238	1575.8563	788.4318	17
12	1321.6681	661.3377	1304.6416	652.8244	1303.6576	652.3324	G	1494.7985	747.9029	1477.7719	739.3896	1476.7879	738.8976	16
13	1434.7522	717.8797	1417.7256	709.3665	1416.7416	708.8745	L	1437.7770	719.3921	1420.7505	710.8789	1419.7664	710.3869	15
14	1505.7893	753.3983	1488.7628	744.8850	1487.7787	744.3930	A	1324.6930	662.8501	1307.6664	654.3368	1306.6824	653.8448	14
15	1576.8264	788.9168	1559.7999	780.4036	1558.8159	779.9116	A	1253.6558	627.3316	1236.6293	618.8183	1235.6453	618.3263	13
16	1663.8585	832.4329	1646.8319	823.9196	1645.8479	823.4276	S	1182.6187	591.8130	1165.5922	583.2997	1164.6082	582.8077	12
17	1794.8989	897.9531	1777.8724	889.4398	1776.8884	888.9478	M	1095.5867	548.2970	1078.5601	539.7837	1077.5761	539.2917	11
18	1851.9204	926.4638	1834.8939	917.9506	1833.9098	917.4586	G	964.5462	482.7767	947.5197	474.2635	946.5356	473.7715	10
19	1922.9575	961.9824	1905.9310	953.4691	1904.9469	952.9771	A	907.5247	454.2660	890.4982	445.7527	889.5142	445.2607	9
20	2070.0259	1035.5166	2052.9994	1027.0033	2052.0154	1026.5113	F	836.4876	418.7475	819.4611	410.2342	818.4771	409.7422	8
21	2183.1100	1092.0586	2166.0834	1083.5454	2165.0994	1083.0534	L	689.4192	345.2132	672.3927	336.7000	671.4087	336.2080	7
22	2296.1941	1148.6007	2279.1675	1140.0874	2278.1835	1139.5954	L	576.3352	288.6712	559.3086	280.1579	558.3246	279.6659	6
23	2383.2261	1192.1167	2366.1995	1183.6034	2365.2155	1183.1114	S	463.2511	232.1292	446.2245	223.6159	445.2405	223.1239	5
24	2454.2632	1227.6352	2437.2366	1219.1220	2436.2526	1218.6300	A	376.2191	188.6132	359.1925	180.0999	358.2085	179.6079	4
25	2511.2847	1256.1460	2494.2581	1247.6327	2493.2741	1247.1407	G	305.1819	153.0946	288.1554	144.5813	287.1714	144.0893	3
26	2612.3323	1306.6698	2595.3058	1298.1565	2594.3218	1297.6645	T	248.1605	124.5839	231.1339	116.0706	230.1499	115.5786	2
27							K	147.1128	74.0600	130.0863	65.5468			1

Supplemental Figure 4. MS/MS-based verification of the S193A mutation in CLPP5S193A-STREPII and comparison to CLPP5-STREPII
(A) Confirmation of the point mutation in the catalytic sites of CLPP5S193A-STREPII by MS/MS of the tryptic peptide (HIRPDVSTVCVGLAAAMGAFLLSAGTK) generated by tryptic digestion of affinity purified CLP complexes. The MS/MS spectrum is from a triply charged precursor ion with m/z of 914.8238 (3^+) with MASCOT ion score of 83 (-5 ppm error) and supports the residue A193. A list of b- and y-ions is listed. This is the same spectrum as shown in Figure 2C.
(B) An example of an MS/MS spectrum of the CLPP5 wild-type peptide (HIRPDVSTVCVGLAASMGAFLLSAGTK) covering the region around S193**.** The MS/MS spectrum is from a triply charged precursor ion with m/z of 920.150193 (3^+) with MASCOT ion score of 126 (0.68 ppm error). The partial peptide sequence listed above the spectrum (GMS$_{193}$AALGL) shown is based on y-ions explaining this reads in reverse order. A list of b- and y-ions is listed.

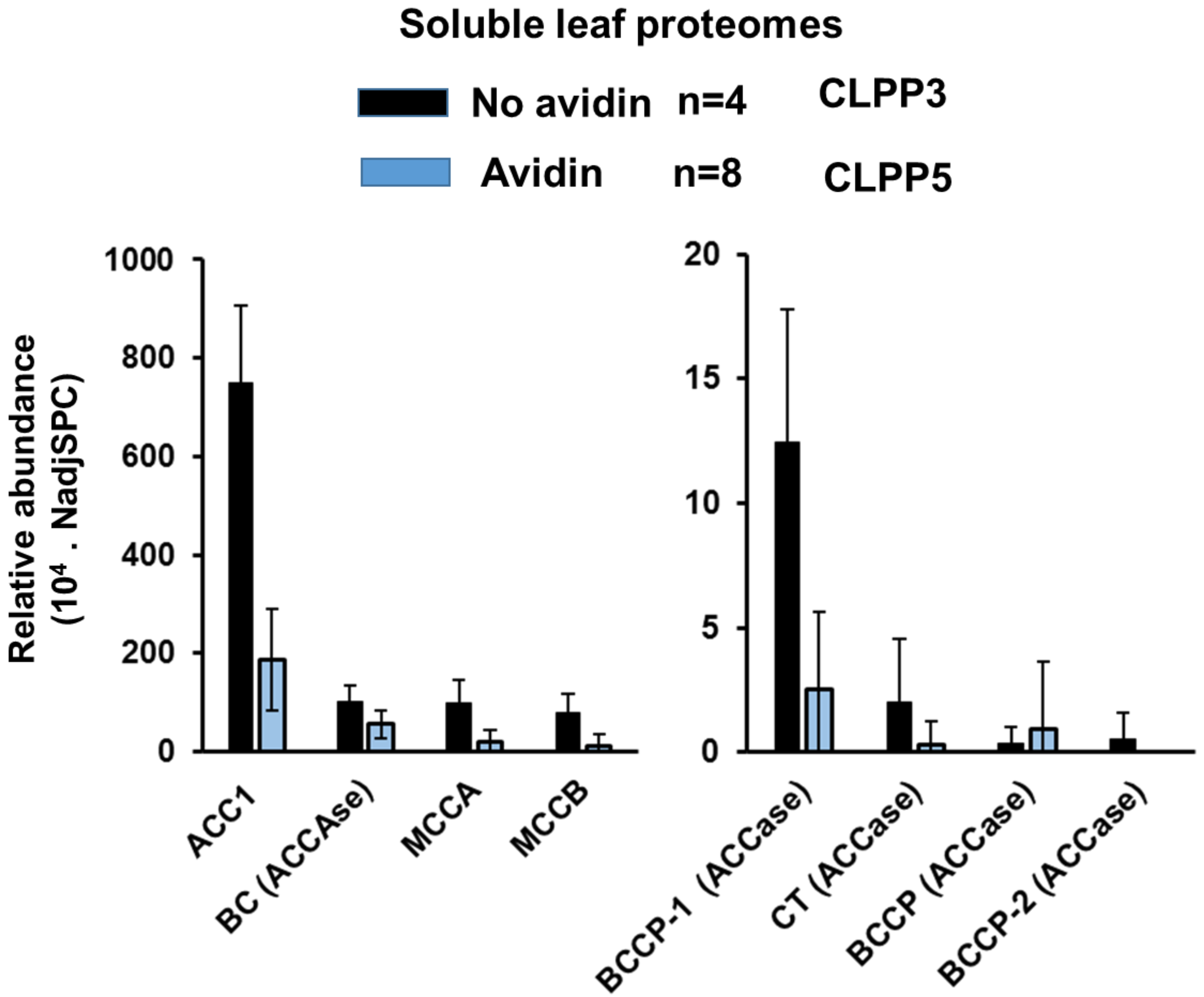

Supplemental Figure 5. Effect of preincubation of soluble leaf proteomes with avidin to reduce the binding and enrichment on streptactin columns for CLPP3S164A-STREPII and CLPP5S193A-STREPII lines. The black bars indicate the abundances of the co-purified biotin-containing proteins from CLPP3S164A-STREPII soluble leaf proteomes without avidin pretreatment. n=4 includes two CLPP3-STREPII and two CLPP3S164A-STREPII lines. The blue bars show those from the avidin-pretreated CLPP5S193A-STREPII soluble leaf proteomes. n=8 includes four CLPP5-STREPII and four CLPP5S193A-STREPII lines. Standard deviations are indicated.
