# Supplementary material for: Consequences of the loss of catalytic triads in chloroplast CLPPR protease core complexes in vivo
Source: Plant Direct. 2018 Oct 25;2(10):e00086. doi: 10.1002/pld3.86 (PMC6508832; doi:10.1002/pld3.86)
Supplement: Supplementary file 4 [file PLD3-2-e00086-s004.pdf]

SUPPORTING INFORMATION

Supplemental Figure 1. Sequence alignment of CLPP3 and CLPP5 with CLPP homologs.
Supplemental Figure 2. Genotyping and RT-PCR analysis of transgenic lines.
Supplemental Figure 3. MS/MS-based verification of the S164A mutation in CLPP3S164A-STREPII and comparison to CLPP3-STREPII
Supplemental Figure 4. MS/MS-based verification of the S193A mutation in CLPP5S193A-STREPII and comparison to CLPP5-WT-STREPII
Supplemental Figure 5. Presence of endogenous biotin containing protein in the streptactin affinity eluates with or without precincubation with avidin
Supplemental Table 1. Primer sets used in this study.
Supplemental Table 2. Protein analysis of streptactin affinity eluates of wt, identified across three affinity experiments with CLPP3-STREPII, CLPP3S164A-STREPII, CLPP5-STREPII and CLPP5S193A-STREPll.

Supplemental Figure 1. Sequence alignment of CLPP3 and CLPP5 with CLPP homologs. Crystalized CLPP homologs from *Helicobacter pylori* (hp), *Escherichia coli* (Ec), *Streptococcus pnneumoniae* (Sp), *Plasmodium falciparum* (Pf), and *Mycobacterium tuberculosis* (Mt) are aligned and compared with CLPP3 and CLPP5 without cTP. The residues in the catalytic triad are indicated with arrowheads. sequence identity (*); high sequence similarity (:); low level of similarity (.)

Supplemental Figure 2. Genotyping and RT-PCR analysis of transgenic lines.
(A) Gene models and the transgenic constructs for complementation. Primers used for genotyping and RT-PCR analysis are numbered (see primer # in Supplemental Table 1). The yellow star highlights the Ser-to-Ala change. The upstream/downstream region and the plastid vector shown are not to scale.
(B) Genotyping for endogenous *CLPP3* (P3; primers P1+P3), transgenic *CLPP3-STREPII* or *CLPP3S164A-STREPII* (P3-STR; primers P4+P5), or the *t-DNA* in *clpp3-1* (primers P1+P2). Eight individual T2 plants from one *CLPP3-STREPII* T1 line in the *clpp3-1* background (upper panel) or *CLPP3S164A-STREPII* T1 line in the *clpp3-1* background (lower panel) were analyzed. wt plants (wt) was used as positive control for *CLPP3* and as the negative control for the *t-DNA*.
(C) RT-PCR analysis of endogenous *CLPP3* and *ACTIN2* (upper panel) and *CLPP3-STREPII* or *CLPP3S164A-STREPll* expression (primer pairs P7+P8 or P6+P9) in the transgenic T1 lines.

Four putative *CLPP3-STREPII* T1 or eight putative *CLPP3S164A-STREPll* T1 plants were analyzed. * - removed from further analysis. wt and *clpp3-1* lines were used as control plants.
(D) RT-PCR analysis of *CLPP5-STREPII* or *CLPP5S193A-STREPll* expression (P14+P9) in the two putative *CLPP5-STREPII* and three putative *CLPP5S193A-STREPII* T1 lines. *ACTIN2* was used as an internal control.

Supplemental Figure 3. **MS/MS-based verification of the S164A mutation in CLPP3S164A-STREPII and comparison to CLPP3-STREPII**
(A) Identification of the point mutation S164 to A164 in the catalytic site of CLPP3S164A-STREPII by MS/MS of the tryptic peptide (ADVSTVCLGLAAAMGAFLLASGSK) generated by tryptic digestion of affinity purified CLP complexes. The MS/MS spectrum is from a doubly charged precursor ion with m/z of 1155.5967 (2^+) with MASCOT ion score of 131 (0.38 ppm error) and supports the residue A164. The partial peptide sequence listed above the spectrum (ALLFAGMA$_{164}$AALGLCV) shown is based on y-ions explaining the reverse order of amino acids. A full list of b- and y-ions is listed. This is the same spectrum as shown in Figure 1D.
(B) An example of an MS/MS spectrum of the CLPP3 wild-type peptide covering the region around S164. MS/MS of the tryptic peptide (ADVSTVCLGLAASMGAFLLASGSK) generated by tryptic digestion of affinity purified CLP complexes. The MS/MS spectrum is from a doubly charged precursor ion with m/z of 1163.5893 (2^+) with MASCOT ion score of 131 (-2.92 ppm error) and supports the residue S164. The partial peptide sequence listed above the spectrum (ALLFAGMS$_{164}$AALGLCV) shown is based on y-ions explaining this reads in reverse order. A full list of b- and y-ions is listed.

Supplemental Figure 4. MS/MS-based verification of the S193A mutation in CLPP5S193A-STREPII and comparison to CLPP5-WT-STREPII
(A) Confirmation of the point mutation in the catalytic sites of CLPP5S193A-STREPII by MS/MS of the tryptic peptide (HIRPDVSTVCVGLAAAMGAFLLSAGTK) generated by tryptic digestion of affinity purified CLP complexes. The MS/MS spectrum is from a triply charged precursor ion with m/z of 914.8238 (3^+) with MASCOT ion score of 83 (-5 ppm error) and supports the residue A193. A list of b- and y-ions is listed. This is the same spectrum as shown in Figure 2C.
(B) An example of an MS/MS spectrum of the CLPP5 wild-type peptide (HIRPDVSTVCVGLAASMGAFLLSAGTK) covering the region around S193**.** The MS/MS spectrum is from a triply charged precursor ion with m/z of 920.150193 (3^+) with MASCOT ion score of 126 (0.68 ppm error). The partial peptide sequence listed above the spectrum

(GMS$_{193}$AALG) shown is based on y-ions explaining this reads in reverse order. A list of b- and y-ions is listed.

Supplemental Figure 5. Effect of preincubation of soluble leaf proteomes with avidin to reduce the binding and enrichment on streptactin columns for CLPP3S164A-STREPII and CLPP5S193A-STREPII lines. The black bars indicate the abundances of the co-purified biotin-containing proteins from CLPP3S164A-STREPII soluble leaf proteomes without avidin pretreatment. n=4 includes two CLPP3-STREPII and two CLPP3S164A-STREPII lines. The blue bars show those from the avidin-pretreated CLPP5S193A-STREPII soluble leaf proteomes. n=8 includes four CLPP5-STREPII and four CLPP5S193A-STREPII lines. Standard deviations are indicated.

Supplemental Table 1. **Primer sets used in this study.**

Supplemental Table 2. Protein analysis of streptactin affinity eluates of wt, identified across three affinity experiments with CLPP3-STREPII, CLPP3S164A-STREPII, CLPP5-STREPII and CLPP5S193A-STREPll.

(A). Direct comparison between proteins identified across three affinity experiments with CLPP3-STREPII, CLPP3S164A-STREPII and wt control (no transgenes) using total soluble leaf extracts. Proteins were annotated based on information in the Plant Proteome Data Base (PPDB). The number of matched adjusted MS/MS spectra (adjSPC) and normalized adjSPC (NadjSPC) are listed. Proteins identified with less than 3 adjSPC across the three experiments were disgarded.

(B) Proteins identified across the 16 affinity experiments with CLPP3-STREPII, CLPP3S164A-STREPII, CLPP5-STREPII or CLPP5S193A-STREPll. Proteins were annotated based on information in the Plant Proteome Data Base (PPDB). The number of matched adjusted MS/MS spectra (adjSPC) and normalized adjSPC are listed. Proteins were identified in at least 3 out of the 16 experiments and with at least four adjSPC across all experiments.
