# Supplementary material for: Consequences of the loss of catalytic triads in chloroplast CLPPR protease core complexes in vivo
Source: Plant Direct. 2018 Oct 25;2(10):e00086. doi: 10.1002/pld3.86 (PMC6508832; doi:10.1002/pld3.86)
Supplement: Supplementary file 5 [file PLD3-2-e00086-s005.pdf]

School of Integrative Plant Science
Plant Biology Section

Cornell University
College of Agriculture and Life Sciences

412 Mann Library
Ithaca, NY 14853-4301
607-255-2131
607-255-5407 (fax)

Ithaca, September 19, 2018

Plant Direct

RE: revision of 2018-00198

Dear Editor,

Thank you for the reviewer's reports and your decision letter for our manuscript **2018-00198** entitled: "**Consequences of the loss of catalytic triads in chloroplast CLPPR protease core complexes *in vivo***" by Jui-Yun Rei Liao, Giulia Friso, Jitae Kim and undersigned.

We have carefully revised the manuscript following the reviewer's comments and suggestions. Please find below a point-by-point response to these reviewers reports. We hope that the revised manuscript is now acceptable for publication in Plant Direct.

Looking forward to your response,

On behalf of all co-authors,

Sincerely,

Klaas van Wijk

Klaas J. van Wijk, Professor
Chair, Section Plant Biology,

Diversity and inclusion are a part of Cornell University's heritage.
We are a recognized employer and educator valuing AA/EEO, protected veterans, and individuals with disabilities.

School for Integrative Plant Sciences (SIPS),
Cornell University, Ithaca, NY;
Tel: 607-2553664; kv35@cornell.edu
http://blogs.cornell.edu/vanwijk/

Reviewer #1:

Minor changes:

Page 3: Before Peltier et al., 2004 there is an "and see" which should be inside the parenthesis or removed.

Removed 'and see'

Page 4: The reference Moreno Beltran et al., 2017 is not correct. The correct version is Moreno et al., 2017 for the paper published in JXB referring to the tobacco RNAi lines.

The reference Moreno et al., 2017 is corrected.

Page 5: The reference is not Moreno Beltran et al., 2017, it should be Moreno et al., 2018.

The reference Moreno et al., 2018 is updated.

Page 6: There is a data not shown here. Could you please provide these results as a supplementary figure.

We removed the statement with negative results about stress treatments, etc. (data not shown)

Page 7: In the chapter corresponding to The catalytic activity of CLPP5 is required for function; S-to-A change in CLPP5 prevents complementation of clpp5-1, you wrote Supplemental Figure 2A,D but later in other pages you write Figure 2B-C. Please be constant and choose the dash or the comma but do not mix them.

We made the style now consistent throughout the manuscript and dashes are changed to comma.

Page 9: In the line 6 of this page you wrote Supplemental Table S2A but then later all the other supplemental material do not have any S preceding the letters. Please be constant and remove the S or add the S to all the other Supplemental data.

S is removed when referring to supplemental tables & figures.

Page 9: Please be constant with the nomenclature for the STREP lines. First time you introduced them as CLPP3-STREPII and CLP3S164A-STREPII. Clearly there is a P missing for the mutant CLP version. So please add one P to that one or delete the extra P in all the other times you wrote CLPP3S164A. Same in line 11 of the same page you just wrote P3-STREPII lines, please change it to CLPP3-STREPII lines as it is written later in line 15 and 19 from the same page. Please review the nomenclature carefully throughout the manuscript.
We reviewed for consistency throughout. The abbreviation of P3/5-STREPII is updated to the CLPP3/5-STREPII; P3S164A is changed to CLPP3S164A-STREPII; P5S193A-STREPII is changed to CLPP5S193A-STREPII.

Page 11: In the last sentence of the page there is a "we" missing or alike.
We inserted 'we'

In the figures 1 and 2 you wrote the letters to designate each panel of the figure in normal case. However, in the legends all of them are capital case which is also the case of Figure 3. Please be consistent and change letters of Figure 1 and 2 to upper case. Same for Supplemental Figure 3 and 4. Please be consistent.
We corrected for consistency, making all panel letters in capital cases.

Besides in Supplemental Figure 2 you have different names for your lines: CLPP5-STR and so on.
We have corrected the naming and made it now fully consistent with the rest of the paper.

Reviewer #2:

The authors concluded that none of those mutant CLP core complexes with reduced catalytic triads allowed for significant accumulation of substrates compared to the fully-active wild-type CLP complex, which seems to be based on the affinity purification experiment unable to detect known CLP substrates. However in the same experiment the authors found some specific proteins (GS2 and FBPA) enriched with the mutant core complexes, both of which seem to meet their criteria for possible candidate substrates. To avoid confusion the discrepancy between this finding and that conclusion needs to be explained or resolved.

Upon further consideration, we removed highlighting these two proteins because i) they are among the most abundant proteins in the chloroplast proteome and identifying these two proteins with just 3-6 MSMS spectra is not sufficient to consider them strong candidates. This change further supports the general message of the paper, that reduction of the number of catalytic triads by 1 or 2 (from 10), does not lead to a detectable trapping of substrates.

Also the authors suggested only a few proteins as candidate substrates, because the other co-purified proteins do not meet their own criteria. However some but not all such proteins also could be potential substrates - perhaps catalytic rates may not be so different between the wild-type and the mutant CLP core complexes, and even the wild-type core might be able to trap the substrates, because the kinetics of those core variants are not determined. I think it worth mentioning or discussing this possibility in the text.

Indeed, is certainly possible that proteins observed in both unmodified CLPP3/5-STREPII and CLPP3/5-mutant-STREPII could be substrates. However, for such proteins there are no good criteria to distinguish between proteins identified in the eluates due to unspecific protein interactions with e.g. the outer surface of the CLP core complex, and proteins residing within the chamber of the proteolytic CLP core because they were selected for degradation. One of the key objectives of the in vivo trapping approach is to obtain a strong candidate list of substrates, such that these can then be further investigated in the context of selection and degradation by the CLP protease system. We mention this specifically now on page 10 (bottom) and 11 (top) in the revised text.

On the other hand their statement regarding structural/functional contribution and physiologic significance (ie. dispensability and essentiality) of the catalytic sites in the different CLP core subunits is consistent with the biochemical analysis and the observed phenotypes of the transgenic plants.

The article is mostly well written and easy to read. The experimentation and analysis for the main figures are described in adequate detail but some minor points are not. The experimental condition for avidin preincubation is not found in the method section.
We have added details for the avidin preincubation in the method section.

The stress experiments also are mentioned in the summary section but only described as "data not shown" in the result and discussion section, and the detailed methods for the stress treatments are also not provided.
Also reviewer #1 mentioned this, and as stated above, we removed this statement from the manuscript.

In addition the description in Supplemental Figure 5 is incomplete (see below).
We added details to this figure legend.

1. Page 9 - line 6: "Supplemental Table S2A" should be Supplemental Table 2A.
Corrected to Supplemental Table 2A.

2. Page 11 - lines 4 to 6: Some reference supporting this notion would be desired (eg. Moreno Beltan et al., Plant Phys, 2017).
The threshold we used here is based on information and ideas from other empirical bacterial trapping studies, as well as our own extensive experience with affinity purifications. We have now added several references to such prior studies. We did not cite Moreno Beltan et al here because the protein samples for MS analysis and the impact of the two approaches are different. Moreno Beltan et al suppressed the whole CLP in vivo by RNAi (losing the intact CLP complex) and analyzed the whole cell proteome timely to minimize the secondary effect. Our approach inactivated the catalytic site (intact CLP complex in vivo) and analyzed the affinity-enriched CLP complexes from different independent lines to remove the secondary effect.

3. Page 11 - lines 26 to 27: Please cite Supplemental Table 2A here to indicate where to see.
Supplemental Table 2A,B are cited in the same paragraph to indicate the data.

4. Page 12 - line 11 to 15: This observation seems not to suggest their specific association with the CLPP5 variants, because THI1 and EF-Tu both were abundantly detected in the affinity purification experiment with the wild type total soluble leaf extracts as well (Supplemental Table 2A).
We agree and we have added this now into the text and reformulated the statement (see page 12).

Substrate trapping could also be verified by immunoblotting comparing accumulation of known substrates (eg. GluTR) in the affinity eluates of STREPII-tagged CLPP3/P5 variants versus non-tagged wild-type plants.
Yes, one could use immunoblotting to test for enrichment of specific proteins, however we wanted to use an unbiased approach and allow for discovery of a wide range of proteins; therefore we used mass spectrometry.

5. Page 14 - line 12: Please remove "Developing siliques."
"Developing siliques" is removed.

6. Page 16 - line 5: Please correct the light/dark cycle condition in the Figure 1A legend.
The light/dark cycle conditions are verified throughout the manuscript and corrected as needed

7. I wonder whether MS/MS spectrum data in main Figures 1d and 2c appear again in Supplemental Figures 3 (a) and 4 (a).
Yes. We strongly believe that is important to provide evidence for the actual point mutation at the protein level in the Figures 1 and 2 in the main paper. Because there is not enough space in the main figures to show also the spectra for the unmodified proteins, we show spectra for both unmodified and mutated proteins in the Supplemental figures 3 and 4. We added a note in the legend of the Supplemental Figures 3 and 4.

8. The description for black and blue bars in the Supplemental Figure 5 is incomplete: please explain in a complete sentence - for example, black bars indicate the abundances of co-purified biotin-containing proteins from CLPP3-STREPII soluble leaf proteomes without avidin pretreatment and blue bars show those from avidin-pretreated CLPP5-STREPII soluble leaf proteomes. The indicated replicate numbers (n=4 and n=8) also need to be adequately explained.
We extended the figure legend following the reviewer suggestions and explained n=4 includes two CLPP3-STREPII and two CLPP3S164A-STREPII and n=8 includes four CLPP5-STREPII and CLPP5S193A-STREPII in the legend.
